# Optic cup morphogenesis requires neural crest-mediated basement membrane assembly

**DOI:** 10.1242/dev.181420

**Published:** 2020-02-21

**Authors:** Chase D. Bryan, Macaulie A. Casey, Rebecca L. Pfeiffer, Bryan W. Jones, Kristen M. Kwan

**Affiliations:** 1 Department of Human Genetics, University of Utah, Salt Lake City, UT 84112, USA; 2 Department of Ophthalmology and Visual Sciences, John A. Moran Eye Center, University of Utah School of Medicine, Salt Lake City, UT 84132, USA

**Keywords:** Eye, Morphogenesis, Extracellular matrix, Basement membrane, Nidogen, Neural crest

## Abstract

Organogenesis requires precise interactions between a developing tissue and its environment. In vertebrates, the developing eye is surrounded by a complex extracellular matrix as well as multiple mesenchymal cell populations. Disruptions to either the matrix or periocular mesenchyme can cause defects in early eye development, yet in many cases the underlying mechanism is unknown. Here, using multidimensional imaging and computational analyses in zebrafish, we establish that cell movements in the developing optic cup require neural crest. Ultrastructural analysis reveals that basement membrane formation around the developing eye is also dependent on neural crest, but only specifically around the retinal pigment epithelium. Neural crest cells produce the extracellular matrix protein nidogen: impairing nidogen function disrupts eye development, and, strikingly, expression of nidogen in the absence of neural crest partially restores optic cup morphogenesis. These results demonstrate that eye formation is regulated in part by extrinsic control of extracellular matrix assembly.

This article has an associated ‘The people behind the papers’ interview.

## Abstract (precis)

**Highlighted Article:** Multidimensional imaging and computational analyses in zebrafish reveal that optic cup morphogenesis is dependent upon neural crest-derived nidogens, which modulate extracellular matrix assembly.

## INTRODUCTION

Vertebrate optic cup morphogenesis requires precise coordination of cell and tissue movements, which generate the shape and organization crucial for eye function (Hilfer, 1983; Schmitt and Dowling, 1994; Schook, 1980; Walls, 1942). Initially, the optic vesicles evaginate from the developing forebrain; the bilayered vesicles will give rise to neural retina and retinal pigment epithelium (RPE). The optic vesicles elongate and their connection to the brain narrows, generating the optic stalk. During invagination, the final stage of optic cup morphogenesis, the optic vesicles bend, become hemispherical, and enwrap the lens as it arises from the overlying ectoderm, while the optic fissure forms along the ventral surface of the optic cup and optic stalk. In zebrafish, optic cup morphogenesis occurs rapidly, at 10-24 h post-fertilization (hpf). Lineage tracing and live imaging, especially in recent work in zebrafish, has enabled detailed analyses of cell movements during eye development, including identification of extended evagination and rim movement (Heermann et al., 2015; Kwan et al., 2012; Li et al., 2000; Picker et al., 2009; Sidhaye and Norden, 2017). Rim movement occurs during invagination: a subset of cells from the medial layer of the optic vesicle moves around the rim of the vesicle and contributes to the lateral layer, the prospective neural retina. Cells remaining in the medial layer flatten and form the RPE. However, the molecular mechanisms governing rim movement and invagination are only partly understood; much work remains to be done to identify extrinsic cues regulating these dramatic cell movements and changes in morphology. Two potential sources of such extrinsic cues are the extracellular matrix (ECM) and nearby cell populations.

The ECM, a crucial regulator of cell adhesion, migration and survival, is implicated in the morphogenesis of many tissues. The ECM acts as a dynamic scaffold, components of which dictate its function with respect to adhesion, rigidity, growth factor presentation, and many other biological processes, but how ECM structures are built and remodeled *in vivo* is not well understood. A complex ECM has long been known to surround the optic vesicle throughout optic cup morphogenesis (Dong and Chung, 1991; Hendrix and Zwaan, 1975; Kwan, 2014; Peterson et al., 1995; Svoboda and O'Shea, 1987; Tuckett and Morriss-Kay, 1986), yet the dynamics of ECM assembly around the developing eye are poorly understood. Only recently have molecular roles of fibronectin and laminin during optic cup morphogenesis been elucidated (Bryan et al., 2016; Hayes et al., 2012; Huang et al., 2011; Ivanovitch et al., 2013; Sidhaye and Norden, 2017); the roles of many other ECM proteins during optic cup morphogenesis are unknown, as are the functions of specialized ECM structures, such as basement membranes (BMs).

The periocular mesenchyme (POM), a heterogeneous cell population comprising neural crest and mesodermally derived mesenchyme, is found in close proximity to the optic cup (Johnston et al., 1979), and multiple tissues in the mature eye are derived in part from these mesenchymal cells (Williams and Bohnsack, 2015). Mesenchymal cells are known to influence the morphogenesis of many epithelial organs through growth factor signaling as well as modifications to the surrounding ECM (Grocott et al., 2011; Nelson and Larsen, 2015; Thesleff, 2003; Wells et al., 2013). Mutations to transcription factors affecting POM development or survival (e.g. *ap2a*, *pitx2* or *zic2*) cause severe optic cup malformations (Bassett et al., 2010; Bohnsack et al., 2012; Gestri et al., 2009; Li and Cornell, 2007; Sedykh et al., 2017), and recent work indicated a role for POM later during optic fissure closure (Gestri et al., 2018). Together, these data suggest a crucial role for the POM in regulating optic cup morphogenesis.

Here, we sought to determine how eye morphogenesis is regulated by a specific subpopulation of the POM, the neural crest. We find that optic cup formation is impaired in mutants exhibiting a severe loss of neural crest, and four-dimensional time-lapse imaging demonstrates that neural crest cells migrate around the eye during optic cup morphogenesis. We pinpoint specific ocular cell movements that are dependent on neural crest: rim movements driving optic cup invagination and RPE cell movements are disrupted in the absence of neural crest. Because mesenchymal cells can act on epithelial organs by modifying the ECM, we used transmission electron microscopy to visualize ECM ultrastructure: in the absence of neural crest, the BM around the eye is disrupted specifically around the RPE. Finally, we uncover a key molecular effector of neural crest interaction with the eye: optic cup morphogenesis is dependent upon nidogens, modulators of ECM assembly, deposited by neural crest cells. Overall, our results reveal how the eye and surrounding tissues cooperate to generate specific ECM structures, which in turn are essential for proper morphogenesis.

## RESULTS

### Optic cup morphogenesis requires neural crest

Previous studies have revealed crosstalk between the developing eye and the neural crest (Bassett et al., 2010; Bohnsack et al., 2012; Eberhart et al., 2008; Grocott et al., 2011; Langenberg et al., 2008; Sedykh et al., 2017), yet we do not understand, from the ocular perspective, what specific aspects of eye development and morphogenesis require this extrinsic cell population. We therefore set out to examine eye morphogenesis in the zebrafish *tfap2a^ts213^;foxd3^zdf10^* double mutant, which displays a near-complete loss of neural crest (Arduini et al., 2009; Wang et al., 2011).

**Fig. 1.**
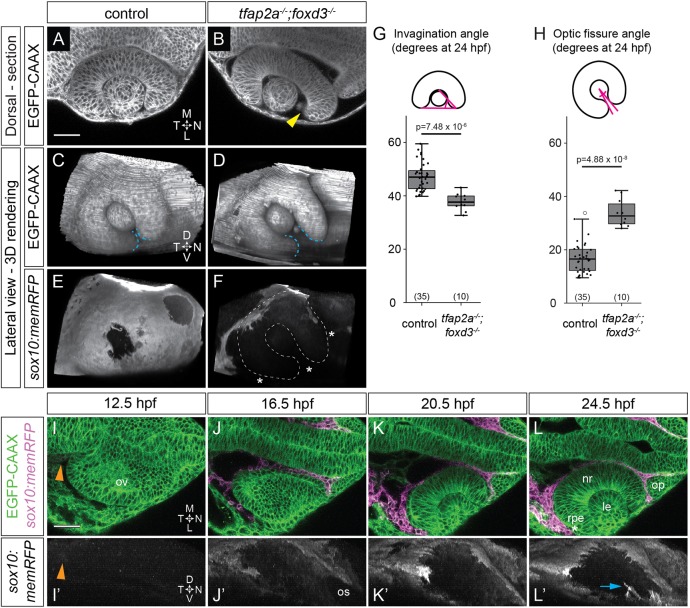
**Optic cup morphogenesis requires neural crest.** (A-F) Dorsal (A,B) and lateral (C-F) views of 24 hpf *Tg(bactin2:EGFP-CAAX);Tg(sox10:memRFP)* double-transgenic control and *tfap2a;foxd3* mutant optic cups. A and B show single confocal sections at the dorsal-ventral midpoint of the lens. Arrowhead in B marks a gap where nasal retina fails to fully enwrap the lens. White dashed lines in F mark the optic cup boundaries. Blue dashed lines (C,D) mark the optic fissure margins. Asterisks in F indicate regions lacking neural crest. (G,H) Quantification of invagination angle (G) and optic fissure angle (H) measured as shown in inset diagrams. *n* (embryos) shown at base of graphs, from three experiments. *P*-values were calculated using one-way ANOVA with Tukey HSD post-hoc test. (I-L′) Time-lapse imaging (12.5-24.5 hpf) of a *Tg(bactin2:EGFP-CAAX);Tg(sox10:memRFP)* double-transgenic embryo. (I-L) Dorsal view, single confocal sections. (I′-L′) Lateral view, 3D rendering of magenta channel from same dataset. Arrowheads in I,I′ indicate RFP^+^ neural crest. Asterisk in L marks RPE between neural crest and neural retina. Arrow in L′ marks neural crest-derived cells entering the optic fissure. Scale bars: 50 μm. D, dorsal; L, lateral; le, lens; M, medial; N, nasal; nr, neural retina; op, olfactory placode; os, optic stalk; ov, optic vesicle; rpe, retinal pigment epithelium; T, temporal; V, ventral.

At 24 hpf, *tfap2a;foxd3* double mutants display gross morphological defects in optic cup invagination and optic fissure formation. At the dorsal-ventral midpoint of the optic cup, the nasal side of the neural retina was flatter than that of sibling controls and failed to completely enwrap the lens (Fig. 1A,B). Quantification of optic cup invagination angle revealed a decrease in the extent to which the retina enwraps the lens in *tfap2a;foxd3* mutants (37.8±1.9°) compared with wild-type controls (47.3±1.8°; Fig. 1G). The optic fissure, a cleft structure at the ventral side of the optic stalk and optic cup, did not form correctly in *tfap2a;foxd3* mutants: control embryos displayed two closely apposed fissure margins with an opening angle of 16.9±1.9°, whereas the optic fissure opening in *tfap2a;foxd3* mutants was larger (33.8±3.1°; Fig. 1C,D,H). Importantly, compared with controls, we found very few *sox10:memRFP^+^* neural crest cells adjacent to the optic cup in *tfap2a;foxd3* double mutants (Fig. 1E,F). In comparison, *tfap2a* and *foxd3* single mutants displayed impaired optic fissure formation and decreased neural crest especially around the ventral portion of the eye (Fig. S1D,E,G,H). However, neither single mutant displayed as severe an effect on invagination angle or optic fissure opening angle as the *tfap2a;foxd3* double mutant (Fig. S1J,K), likely due to the presence of more neural crest cells.

We also assayed optic cup development in *alyron^z24^* (*paf1*) mutants in which neural crest development is severely impaired in an independent manner (Cretekos and Grunwald, 1999). Invagination was more severely disrupted in *paf1* mutants (25.6±3.6°) compared with wild-type controls or *tfap2a;foxd3* double mutants (Fig. S1C,J). Optic fissure formation was so severely affected that optic fissure margins could not be defined, nor measured (Fig. S1F). Similar to the *tfap2a;foxd3* mutants, *sox10:memRFP^+^* cells surrounding the eyes of *paf1* mutants were substantially reduced at 24 hpf (Fig. S1I; Fig. 1F). However, as *paf1* is expressed ubiquitously (Nguyen et al., 2010; Thisse and Thisse, 2004), despite a severe loss of neural crest, it is possible that *paf1* also plays an intrinsic role in optic cup development. Thus, further analysis of neural crest function in optic cup morphogenesis was carried out solely on *tfap2a;foxd3* double mutants.

Previous studies have suggested a role for POM in optic fissure closure (Gestri et al., 2018; Hero, 1990; Hero et al., 1991; James et al., 2016; Lupo et al., 2011; Weiss et al., 2012). Consistent with these data, most *tfap2a;foxd3* double mutants harbored optic fissure defects and gaps in ocular pigmentation (indicative of coloboma) at 52 hpf (59.38% versus 7.62% of control embryos; Fig. S1L,M). Although these previous studies focused largely on optic fissure fusion, which occurs after optic cup morphogenesis, our observations at 24 hpf demonstrate that earlier stages of optic cup morphogenesis are also dependent upon neural crest.

### The developing eye is in close proximity to neural crest throughout optic cup morphogenesis

In zebrafish, optic cup morphogenesis occurs at 10-24 h hpf, during which time the optic vesicles evaginate and undergo stereotypical movements and shape changes to become the organized optic cup, comprising neural retina, RPE and lens. Neural crest cells have previously been observed migrating near the eye during these stages of optic cup morphogenesis, but the extent of the interactions between these tissues is unknown (Gestri et al., 2018; Langenberg et al., 2008). To determine how optic cup morphogenesis might be influenced by neural crest, we first asked when and where these cells interact with the developing eye. To visualize both the eye and neural crest, we used double-transgenic *Tg(bactin2:EGFP-CAAX)^z200^;Tg(sox10:memRFP)^vu234^* zebrafish in which GFP labels all cell membranes and RFP labels neural crest cell membranes (Gordon et al., 2018; Kirby et al., 2006). We performed four-dimensional time-lapse imaging of optic cup morphogenesis (12.5-24.5 hpf; Fig. 1I-L′, Movies 1 and 2). At the start of our imaging, neural crest cells had made contact with the posterior margin of the optic vesicle (Fig. 1I,I′). As the optic vesicle elongated, neural crest cells migrated anteriorly between the prospective brain and optic vesicle, around to the nasal margin of the optic vesicle (Fig. 1J,J′, Movie 1), then enwrapped the optic stalk (Fig. 1J′, Movie 2). During invagination, neural crest cells migrated laterally and ventrally to surround the posterior and ventral optic cup, and appeared to be in close contact with the eye, more so than the prospective brain (Fig. 1K,K′). By 24.5 hpf, when the optic fissure had formed, neural crest moved through the fissure into the space between the neural retina and lens (Fig. 1L′, arrow). By the end of the process, the RPE side of the optic cup was encapsulated by neural crest (Fig. 1L).

### Retina and RPE cell movements require neural crest

Owing to the morphological defects in the nasal and ventral optic cup in *tfap2a;foxd3* double mutants, we initially hypothesized that optic cup patterning might depend upon neural crest. Antibody staining for Pax2a, a transcription factor expressed in the ventral optic cup and optic stalk (Fig. S2A), revealed that Pax2a expression is expanded dorsally and temporally in *tfap2a;foxd3* mutants (Fig. S2D,D′,I; 134.2±10.5° compared with 100.3±5.9° in sibling controls), but, interestingly, only in the RPE layer (Fig. S2D,D′, arrowheads). This was observed in *tfap2a;foxd3* double mutants as well as *tfap2a* and *foxd3* single mutants (Fig. S2B-C′). In other models of optic cup mispatterning, Pax2a is expanded not just into the RPE layer but throughout the ventral retina (Lee et al., 2008; Sedykh et al., 2017). The *tfap2a;foxd3* mutant phenotype is distinct in its restriction to the RPE layer, and suggests that cell movements may be disrupted.

**Fig. 2.**
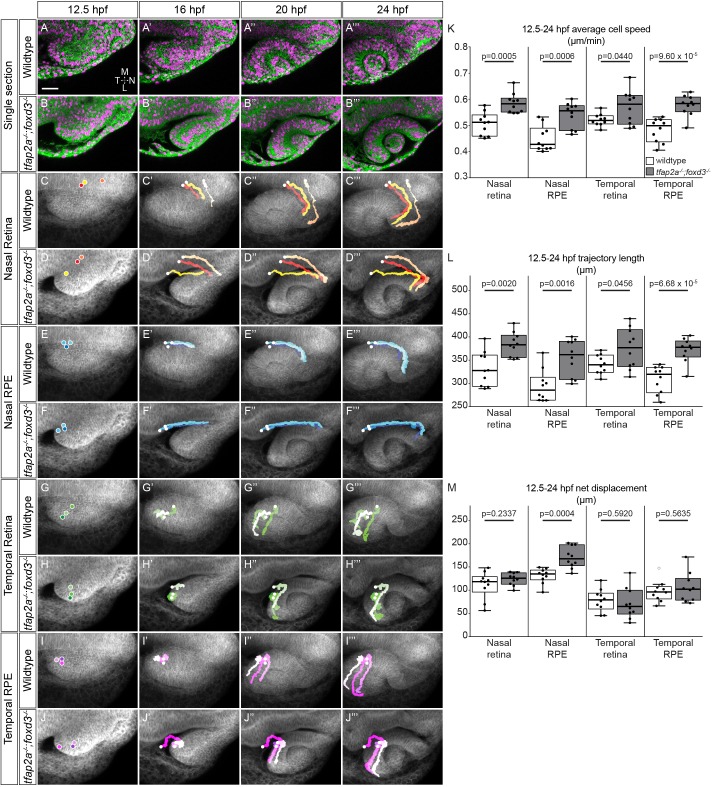
**Optic cup cell movements are disrupted in *tfap2a;foxd3* mutants.** (A-J‴) Time-lapse imaging (12.5-24 hpf) of optic cup morphogenesis of wild-type and *tfap2a;foxd3* mutant embryos. (A-B‴) Dorsal view, single confocal sections from wild-type (A-A‴) and *tfap2a;foxd3* mutant (B-B‴) 4D datasets. EGFP-CAAX (green) labels cell membranes; H2A.F/Z-mCherry (magenta) labels nuclei. (C-J‴) Average projections of EGFP-CAAX channel (gray) with indicated nuclear trajectories overlaid. Trajectories were generated by accumulating nuclear selections over time. (K-M) Average 3D cell speed (K), total 3D trajectory length (L) and 3D net displacement (M) of cells contributing to each region at 24 hpf. *n*=10 cells from each region (two embryos/genotype, five cells each). *P*-values were calculated using Welch's *t*-test. Scale bar: 50 µm. L, lateral; M, medial; N, nasal; T, temporal.

We hypothesized that specific optic cup morphogenetic movements are dependent on neural crest; uncovering these would allow us to pinpoint the cellular basis of eye defects in the absence of neural crest, and also identify specific regions of the eye where neural crest might influence development. Based on the gross morphological phenotype and Pax2a localization, we suspected that rim movement might be disrupted in *tfap2a;foxd3* mutants. During invagination, a subset of cells executes rim movement, moving from the medial layer around the rim of the developing optic cup into the lateral layer to contribute to neural retina (Heermann et al., 2015; Kwan et al., 2012; Picker et al., 2009; Sidhaye and Norden, 2017). The expanded Pax2a expression we observed could result from failure of Pax2a^+^ cells to undergo rim movement into the neural retina, thus remaining in the apparent RPE layer. In addition, we hypothesized that RPE cell movements might also depend on neural crest, given their proximity throughout optic cup morphogenesis (Fig. 1I-L). To determine if specific regional eye cell movements are dependent on neural crest, we performed live imaging of optic cup morphogenesis in wild-type and *tfap2a;foxd3* mutant embryos followed by four-dimensional cell tracking using LongTracker (Kwan et al., 2012) and quantitative analysis of 3D speed and 3D trajectory (Fig. 2, Movie 3).

We first examined movements of nasal retina and RPE cells because we saw gross morphological defects in the nasal hemisphere of *tfap2a;foxd3* double-mutant eyes. We selected these cells from their final positions at 24 hpf in double-mutant and wild-type data sets (Gordon et al., 2018), and tracked these cells retrospectively to establish their origins and movements from the optic vesicle stage; a subset of cells and trajectories is shown (Fig. 2). Nasal retina cells originated from a similar location in wild type and *tfap2a;foxd3* mutants (Fig. 2C-D‴, Movies 4 and 5). The trajectory shape was similar, but the timing was altered: wild-type cells turned away from the midline at ∼17.75 hpf; however, that turn was delayed in *tfap2a;foxd3* mutants until ∼19.5 hpf (Movies 4 and 5). Quantification of these movements in three dimensions (Fig. 2K-M) revealed that nasal retina cells unexpectedly move faster and farther in *tfap2a;foxd3* mutants (average speed 0.60±0.04 μm/min; total distance 402.80±17.33 μm) than in wild types (average speed 0.53±0.03 μm/min; total distance 352.41±29.99 μm; Fig. 2K,L), although net displacement was unchanged (wild type 111.55±18.18 μm; *tfap2a;foxd3* 124.08±8.43 μm; Fig. 2M). Nasal RPE cell movements were also altered in *tfap2a;foxd3* mutants. These cells originate from the same domain in wild-type and mutant embryos, but trajectory shape was altered: wild-type nasal RPE cells moved along an arc-like trajectory to the nasal RPE margin; however, *tfap2a;foxd3* mutant nasal RPE cells traveled along a straight anterior path until 19.5 hpf, when they made a sudden 90° turn toward the optic cup margin (Fig. 2E-F‴, Movies 6 and 7). As with the nasal retina, nasal RPE cells moved faster and farther in *tfap2a;foxd3* mutants (average speed 0.57±0.02 μm/min; total distance 385.75±15.71 μm) than in wild types (average speed 0.45±0.05 μm/min; total distance 303.01±34.06 μm; Fig. 2K,L). Additionally, net displacement of nasal RPE cells was increased in *tfap2a;foxd3* mutants (170.76±14.55 μm) compared with wild types (131.17±10.16 μm; Fig. 2M). These data suggest that cell motility within the nasal optic cup is restricted by neural crest; without nearby neural crest, cells in the nasal retina and RPE move faster and farther and with incorrect timing.

Although we did not see obvious morphological abnormalities on the temporal side of the eye in *tfap2a;foxd3* double mutants, we wondered if cell movements here might also be defective; this region of the eye is the first to contact neural crest (Fig. 1). In wild-type embryos, temporal retina cells exhibiting rim movement travel around the optic cup rim starting at ∼20 hpf (Fig. 2G-G‴, Movie 8). In *tfap2a;foxd3* mutants, equivalent cells did not initiate rim movement until ∼22 hpf (Fig. 2H-H‴, Movie 9). As with nasal retina cells, the 3D speeds and distances were increased in *tfap2a;foxd3* mutants (average speed 0.57±0.04 μm/min; total distance 375.01±27.53 μm) compared with wild types (average speed 0.52±0.01 μm/min; total distance 341.35±12.86 μm; Fig. 2K,L), whereas net displacement remained unchanged (wild type 78.50±15.07 μm; *tfap2a;foxd3* 71.48±20.79 μm; Fig. 2M). We also examined temporal RPE cells, which are close to neural crest throughout most of optic cup morphogenesis. Temporal RPE cell trajectories appeared similar between wild type and *tfap2a;foxd3* mutants (Fig. 2I-J‴, Movies 10 and 11); however, cell motility was again altered. Similar to the temporal retina, temporal RPE cells moved faster and farther in *tfap2a;foxd3* mutants (average speed 0.58±0.02 μm/min; total distance 372.07±16.21 μm) compared with wild types (average speed 0.48±0.03 μm/min; total distance 309.06±18.20 μm; Fig. 2K,L). Unlike the nasal RPE, net displacement was unchanged for temporal RPE cells (wild type 97.69±14.15 μm; *tfap2a;foxd3* 104.88±19.83 μm; Fig. 2M). Despite a lack of gross morphological defects in the temporal optic cup in *tfap2a;foxd3* mutants, cell tracking uncovers abnormal temporal retina and RPE cell movements.

The medial side of the developing optic cup, which is in close proximity to neural crest, gives rise to RPE and nasal and temporal retina margins. Taken together, we find that cell movements executed by these different eye domains are abnormal when neural crest is lost. Specifically, 3D cell speed and distance traveled are altered: cells move faster and farther in the absence of neural crest. Additionally, the defects in nasal and temporal rim movement in *tfap2a;foxd3* double mutants may explain the Pax2a expansion into the RPE. We conclude that cell movements in the optic cup require neural crest, and their disruption in *tfap2a;foxd3* mutants results in impaired RPE and rim movement.

### BM formation is disrupted specifically around the RPE in *tfap2a;foxd3* mutants

Having demonstrated a role for neural crest in regulating cell movements, we sought to determine the underlying mechanism by which neural crest influences the eye. Previous work suggested that POM signals to the developing eye via TGFβ ligands (Fuhrmann et al., 2000; Grocott et al., 2011). Using an antibody against phospho-Smad3 to detect sites of active TGFβ signaling, we investigated whether loss of neural crest alters TGFβ signaling within the optic cup. We did not, however, detect any differences in phospho-Smad3 localization between control and *tfap2a;foxd3* mutant eyes, suggesting that neural crest is dispensable for TGFβ signaling in the zebrafish optic cup at 24 hpf (Fig. S2E-H, quantified in Fig. S2J,K).

**Fig. 3.**
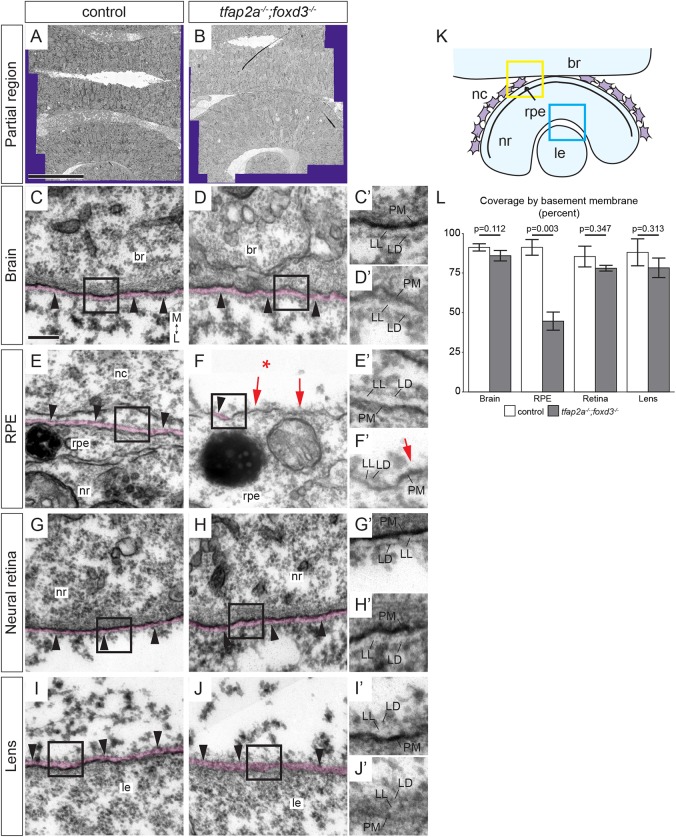
**The basement membrane around the RPE is disrupted in *tfap2a;foxd3* mutants.** (A,B) Tiled arrays of 2 nm/pixel resolution transmission electron micrographs of eyes and forebrains of 24 hpf wild-type control (A) and *tfap2a;foxd3* mutant (B) zebrafish. Acquired regions extend across the brain and through the opposite eye. Purple area is the boundary of captured images. (C-J′) Basement membranes around the brain, RPE, neural retina, and lens in control (C,E,G,I) and *tfap2a;foxd3* mutant (D,F,H,J) embryos imaged at high resolution (∼0.5 nm/pixel). BMs are pseudocolored magenta, and black boxes are enlarged in C′-J′ to aid visualization. The BM around the RPE of *tfap2a;foxd3* mutant embryos appears to be discontinuous (F,F′ red arrows) compared with wild type (E,E′); other BMs appear normal and continuous (black arrowheads). Asterisk in F indicates absence of neural crest. (K) Diagram of tissues imaged. Boxes indicate brain/neural crest/RPE (yellow) and neural retina/lens (blue) regions re-imaged at high resolution (∼0.5 nm/pixel). (L) Quantification of the percentage of tissue surface lined with discernable, continuous BM containing both lamina lucida and lamina densa. *P*-values were calculated using Welch's *t*-test; error bars represent ±2×s.e.m. Scale bars: 50 µm (A), 200 nm (C). Magnification: 10,000× (C-J); 20,000×(C′-J′). All images transverse sections, anterior views. br, brain; L, lateral; LD, lamina densa; le, lens; LL, lamina lucida; M, medial; nc, neural crest; nr, neural retina; PM, plasma membrane; rpe, retinal pigment epithelium.

Data from other organ systems demonstrate that mesenchymal cells can regulate epithelial morphogenesis via modifications to the ECM. We therefore asked if periocular ECM might be defective when neural crest is lost. Laminin-1 and fibronectin appeared to be unaltered between wild types and *tfap2a;foxd3* double mutants (Fig. S3, quantified in Fig. S3I,J). Despite these core ECM components being present, mesenchymal cells can also regulate the ECM via assembly of matrices into a BM. Using transmission electron microscopy (TEM), we directly visualized BMs at the basal surfaces of the brain, RPE, neural retina, and lens in 24 hpf wild types and *tfap2a;foxd3* mutants (Fig. 3A,B). The BM lines the dark plasma membrane, and appears as a thin, contiguous surface comprising the lighter and thicker lamina lucida and darker, thin lamina densa (Bader et al., 2013; Feitosa et al., 2011; Yurchenco and Schittny, 1990). We observed a well-defined BM lining the brain in both *tfap2a;foxd3* double mutants and control embryos; BM covers the brain over ∼91% in control and ∼86% in *tfap2a;foxd3* double mutants (Fig. 3C-D′,L). The BMs lining the neural retina and lens (Fig. 3G-J′), a region in which no neural crest migrates during this process, also appeared to be intact in *tfap2a;foxd3* mutants compared with wild-type controls, with BM covering ∼85% of the retina and ∼88% of the lens in controls, and ∼78% of the retina and 78% of the lens in *tfap2a;foxd3* double mutants (Fig. 3L). In contrast, the BM surrounding the RPE was defective in *tfap2a;foxd3* double mutants (Fig. 3F,F′, arrows): in *tfap2a;foxd3* mutants, only ∼45% of the RPE displayed a BM with both lamina lucida and lamina densa discernable, whereas controls displayed ∼91% coverage by BM (Fig. 3E,E′, arrowheads, 3L). Given that neural crest is in close proximity to the developing RPE throughout eye morphogenesis and no other BMs around the eye are disrupted in *tfap2a;foxd3* mutants, these results suggest that neural crest contributes to BM development or stability specifically around the RPE. To our knowledge, this is the first indication that neural crest influences the BM around the developing eye, and this may represent a novel mechanism for regulating eye morphogenesis.

### The BM protein nidogen is produced in part by neural crest and is decreased in *tfap2a;foxd3* mutants

**Fig. 4.**
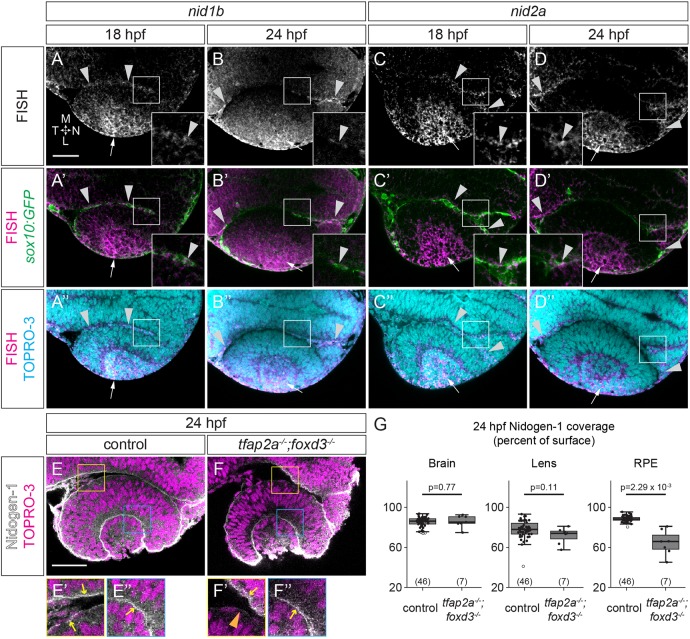
**Nidogen mRNA and protein are expressed around the developing eye.** (A-D″) Fluorescence *in situ* hybridization (FISH) for *nid1b* (A,B) and *nid2a* (C,D) in *Tg(sox10:GFP)* embryos. (A′-D′) FISH (magenta) merged with *sox10:GFP* expression (green) to visualize colocalization between FISH and GFP^+^ neural crest (arrowheads). (A″-D″) FISH merged with nuclei (TO-PRO-3, cyan). Arrows mark lens expression. (E-F″) Immunofluorescence for nidogen 1. In 24 hpf control embryos, nidogen 1 protein (grayscale) is detected along the brain and RPE (yellow box in E, arrows in E′) and lens-retina interface (blue box in E, arrow in E″). In 24 hpf *tfap2a;foxd3* mutants, nidogen 1 is discontinuous around the RPE (F′, orange arrowhead), but still lines the brain (F′, arrow) and lens-retina interface (F″, arrow). (G) Measurements of nidogen 1 protein coverage around the brain, lens and RPE. Percentages were calculated by dividing the length of antibody labeling over the total tissue surface length. *P*-values calculated using Welch's *t*-test. *n* (embryos) shown at base of graphs. Scale bars: 50 µm. Dorsal view, single confocal sections. L, lateral; M, medial; N, nasal; T, temporal.

To understand the underlying cause of BM disruption around the RPE in the *tfap2a;foxd3* double mutant, we set out to identify individual ECM components that might be affected. Nidogen is an ECM crosslinking protein crucial for BM assembly (Ho et al., 2008), which, together with laminin-1, is capable of supporting embryonic stem cell-derived optic cup morphogenesis *in vitro* (Eiraku et al., 2011). Nidogen, provided by mesenchymal cells, is necessary for mouse lung and kidney epithelial morphogenesis (Bader et al., 2005; Ekblom et al., 1994; Kadoya et al., 1997; Willem et al., 2002). Nidogen expression is found in mouse POM and invaginating lens (Dong and Chung, 1991), and zebrafish data suggest that *nidogen 1a* (*nid1a*), *nidogen 1b* (*nid1b*) and *nidogen 2a* (*nid2a*) are expressed near the eye during optic cup morphogenesis (Carrara et al., 2019; Kudoh et al., 2001; Thisse and Thisse, 2004; Zhu et al., 2017). First, we performed our own whole-mount *in situ* hybridization for the four zebrafish nidogen genes (Fig. S4); at the stages examined, *nid1a* was expressed primarily in developing somites (Fig. S4A,E,I), whereas *nid2b* was detected diffusely throughout the head at a low level (Fig. S4D,H,L). In contrast, *nid1b* and *nid2a* were expressed in a pattern suggestive of POM (Fig. S4B,C,F,G,J,K). Because these specific periocular cell populations were not clearly defined in whole-mount *in situ* hybridization, we visualized expression of the nidogen genes at cellular resolution. Fluorescence *in situ* hybridization in *Tg(sox10:GFP)^ba4^* transgenics (Dutton et al., 2008) revealed that both *nid1b* and *nid2a* are expressed by *sox10:GFP*^+^ neural crest cells around the developing optic cup at 18 and 24 hpf (Fig. 4A-D″). *nid1b* and *nid2a* were also expressed in the ectoderm and developing lens at these time points, but notably appeared to be absent in the neural retina and RPE.

As confirmation that nidogens are produced by neural crest, we examined nidogen 1 protein localization around the eye, and whether its deposition is reduced in *tfap2a;foxd3* double mutants; although we tested nidogen 2 antibodies in zebrafish, we were unable to achieve specific staining, so our analysis is limited to nidogen 1. In 24 hpf wild-type embryos, nidogen 1 protein was found at basal surfaces surrounding the RPE, neural retina, and lens (Fig. 4E-E″), consistent with localization to BMs around the eye, as well as the brain. Nidogen 1 protein was found deposited along a similar percentage of brain and lens surface in *tfap2a;foxd3* mutants (brain ∼86.0%, lens ∼71.2%) and wild-type siblings (brain ∼85.2%, lens ∼77.8%; Fig. 4G), but was diminished around the RPE of *tfap2a;foxd3* mutants (Fig. 4F-G). Whereas nidogen lined an average of 88.4% of the RPE surface in 24 hpf wild-type embryos, only ∼64.7% of the RPE was lined in *tfap2a;foxd3* mutants. Notably, much of the nidogen signal surrounding the RPE in the double mutant was found closer to the nasal and temporal margins of the optic cup, where the RPE is in close proximity to the olfactory placode and surface ectoderm, respectively, which provide other sources of nidogen production (Fig. 4F-F″). Taken together, these data suggest that neural crest produces a portion of the nidogen around the RPE, and thus we hypothesize that BM assembly around the eye is a multi-tissue process: nidogen, partially supplied by neural crest, may be necessary for BM assembly specifically around the RPE.

### Dominant-interfering nidogen disrupts optic cup morphogenesis

The discontinuous BM we observed around the RPE in the *tfap2a;foxd3* double mutant was reminiscent of BM alterations found when nidogen is disrupted (genetically or functionally) in mouse lung and kidney morphogenesis (Bader et al., 2005; Ekblom et al., 1994; Kadoya et al., 1997; Willem et al., 2002). This observation, coupled with the finding that *nid1b* and *nid2a* are expressed by neural crest cells, suggested a potential functional role for nidogen in optic cup morphogenesis, which we set out to test.

**Fig. 5.**
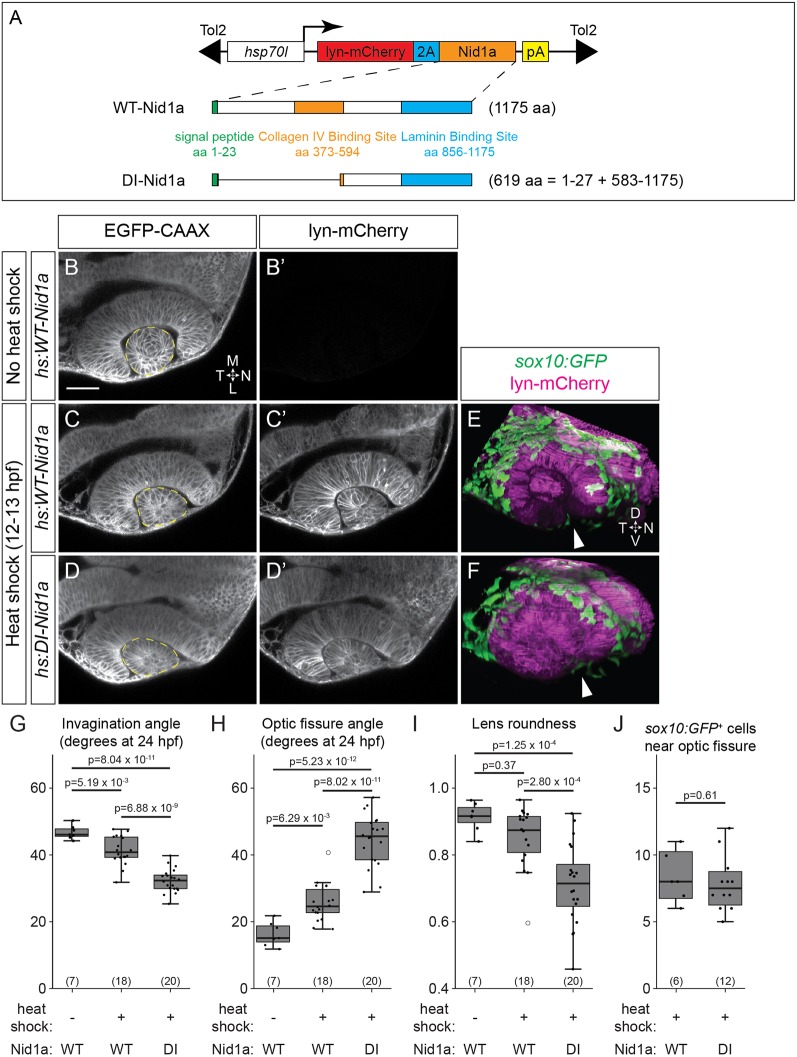
**Dominant-interfering nidogen disrupts optic cup morphogenesis.** (A) Schematics of full length (WT) and dominant-interfering (DI) zebrafish nidogen 1a and transgene constructs. (B-D′) *Tg(bactin2:EGFP-CAAX)* transgenics also carrying either *hs:WT-Nid1a* (B-C′) or *hs:DI-Nid1a* (D,D′) transgenes. Control embryos (B), not heat shocked, are lyn-mCherry^−^ (B′); experimental embryos were heat shocked at 12-13 hpf (C-D′). Dorsal view, single confocal sections. (E,F) *Tg(sox10:GFP)^+^;hs:WT-Nid1a* (E) or *hs:DI-Nid1a* (F) transgenics heat shocked at 12-13 hpf. GFP^+^ neural crest cells migrate around the optic cup and into the optic fissure (arrowheads) in both conditions. Lateral view, 3D renderings, 24 hpf. (G-J) Quantification of invagination angle (G), optic fissure angle (H), lens roundness (I) and *sox10:GFP*^+^ cell number at the optic fissure (J). Dashed lines in B-D mark lens outlines used for roundness measurements. *sox10:GFP*^+^ cells were counted as near the optic fissure if they were located in the optic fissure or were in contact with a margin of the optic fissure. *n* (embryos) shown at base of graphs, from one to three experiments. *P*-values were calculated using one-way ANOVA with Tukey HSD post-hoc test (G-I) or Welch's *t*-test (J). Scale bar: 50 μm. D, dorsal; L, lateral; M, medial; N, nasal; T, temporal; V, ventral.

Because multiple nidogens are expressed in the neural crest and head during eye development, we initially took a dominant-interference strategy to disrupt nidogen function. We used Nd-III, an internal deletion form of mouse nidogen 1 that lacks the domains required for binding collagen IV and heparin sulfate proteoglycan, but retains the laminin-binding domain (Fox et al., 1991; Reinhardt et al., 1993). *In vitro* characterization demonstrated that Nd-III acts in a dominant-inhibitory fashion by competitively preventing full-length nidogen from binding to laminin, thereby blocking the bridging of laminin to other ECM components (Pujuguet et al., 2000). Zebrafish Nidogen 1a is most closely related to mouse nidogen 1; thus, we generated stable transgenic zebrafish lines expressing either full-length zebrafish *nid1a* (Fig. 5A, WT-Nid1a), or dominant-inhibitory *nid1a* based on the Nd-III fragment (Fig. 5A, DI-Nid1a). Using heat-shock inducible transgenes, we were able to both temporally control expression of WT-Nid1a or DI-Nid1a and detect transgene expression via the lyn-mCherry reporter (Fig. 5A).

We observed neural crest cells contacting the optic vesicle at 12.5 hpf (Fig. 1I); therefore, we heat shocked at 12-13 hpf to induce WT-Nid1a or DI-Nid1a expression at the onset of neural crest migration around the optic vesicle. We examined optic cups of *Tg(bactin2:EGFP-CAAX)* zebrafish double transgenic for *Tg(hs:WT-Nid1a)* or *Tg(hs:DI-Nid1a)* (Fig. 5B-D′). Ubiquitous overexpression of WT-Nid1a slightly but significantly impaired invagination (42.3±1.7° versus 46.8±1.4° in controls), optic fissure formation (25.7±2.6° versus 16.3±2.5° in controls; Fig. 5C,C′,G,H) and lens morphogenesis, resulting in a slightly ovoid lens (Fig. 5I). However, DI-Nid1a overexpression substantially impaired optic cup morphogenesis, including invagination (32.3±1.5°), optic fissure formation (44.5±3.4°) and lens morphogenesis (Fig. 5D,D′,G-I). This is consistent with a recent study using morpholinos and RNA injection of an inferred dominant-negative form of Nidogen 1b (Carrara et al., 2019). Because such dramatic phenotypes could be due to indirect effects on neural crest migration, we visualized neural crest in the *sox10:GFP* transgenic line when either WT-Nid1a or DI-Nid1a was overexpressed (Fig. 5E,F). As neural crest cells migrate into the optic fissure near the end of optic cup morphogenesis (Fig. 1L′), we quantified the number of GFP^+^ cells in contact with the optic fissure at 24 hpf as a measure of migration. We found that heat shock-induced overexpression of WT-Nid1a or DI-Nid1a does not affect neural crest migration (Fig. 5J) and therefore conclude that DI-Nid1a impairs optic cup morphogenesis through direct effects on the eye.

### Nidogen overexpression partially rescues *tfap2a;foxd3* mutant optic cup phenotypes

Nidogen expression in the neural crest, coupled with phenotypes observed when DI-Nid1a is overexpressed, suggested that disruptions to nidogen's matrix-bridging function might underlie the eye morphogenesis defects in *tfap2a;foxd3* double mutants. We therefore asked whether those defects might be due to a lack of nidogen deposited by neural crest, and whether we might be able to rescue aspects of eye morphogenesis by providing nidogen.

**Fig. 6.**
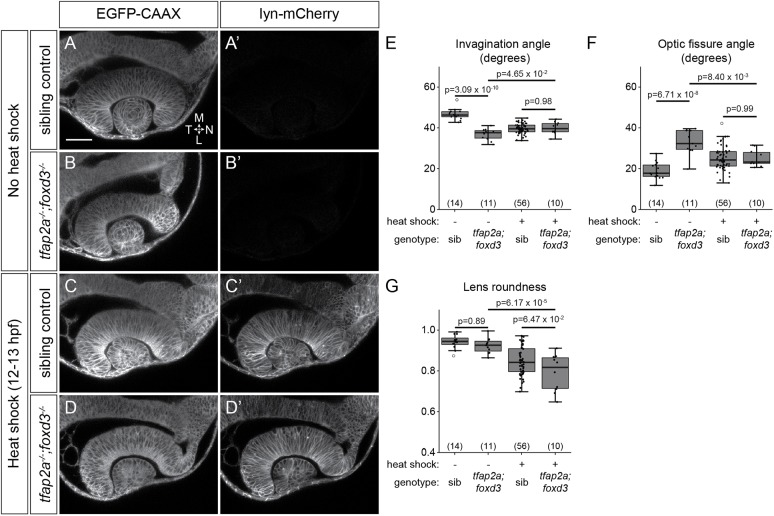
**Nid1a overexpression partially rescues optic cup morphogenesis in *tfap2a;foxd3* mutants.** (A-D′) Double-transgenic embryos [*Tg(bactin2:EGFP-CAAX);Tg(hs:WT-Nid1a)*]: sibling control and *tfap2a;foxd3* mutant embryos not heat shocked (A-B′), or heat shocked at 12-13 hpf (C-D′). Dorsal view, single confocal sections, 24 hpf. (E-G) Quantification of invagination angle (E), optic fissure angle (F) and lens roundness (G). *n* (embryos) shown in the base of the graph, from two or three experiments. *P*-values were calculated using one-way ANOVA with Tukey HSD post-hoc test. Scale bar: 50 μm. L, lateral; M, medial; N, nasal; T, temporal.

To test this, we used our heat shock-inducible transgenic to ubiquitously overexpress WT-Nid1a in *tfap2a;foxd3* double mutants, in which there would be no neural crest present around the RPE to produce nidogen. We generated *Tg(bactin2:EGFP-CAAX);Tg(hs:WT-Nid1a)* double-transgenic *tfap2a;foxd3* double mutants, to visualize optic cups of control (non-heat-shocked) and heat-shocked embryos. Control *tfap2a;foxd3* mutants, as expected, exhibited retinal invagination and optic fissure formation defects (Fig. 6A-B′,E,F). As described (Fig. 5), overexpression of WT-Nid1a in wild types slightly impairs invagination, optic fissure formation and lens morphogenesis (Fig. 6C,C′,E-G). Upon WT-Nid1a overexpression in *tfap2a;foxd3* double-mutant embryos, mutant phenotypes appeared to be partially rescued: the nasal retina more fully enwrapped the lens and the optic fissure margins were more closely set (Fig. 6D,D′,E-G). Quantification revealed that WT-Nid1a overexpression improves invagination angle (39.9±1.8° versus 36.8±1.6° in control double mutants; Fig. 6E) and optic fissure formation (25.1±2.4° versus 33.0±3.5° in control double mutants; Fig. 6F). The phenotype of heat-shocked mutant embryos was very similar to that of heat-shocked wild-type embryos, with no statistical difference in invagination angle (wild type 39.6±0.7°; *tfap2a;foxd3* 39.9±1.8°; Fig. 6E) or optic fissure angle (wild type 25.3±1.6°; *tfap2a;foxd3* 25.1±2.4°; Fig. 6F). Lenses in both genotypes were similarly affected, becoming ovoid (Fig. 6G). These data suggest that ubiquitous expression of nidogen can partially rescue optic cup morphogenesis in the absence of neural crest. Despite this, our observation of the overexpression phenotype, as opposed to a full rescue, was slightly concerning as it demonstrated that ectopic nidogen can interfere with optic cup morphogenesis, but did not fully establish whether it plays a vital role in the process.

### Nidogen is necessary for optic cup morphogenesis

**Fig. 7.**
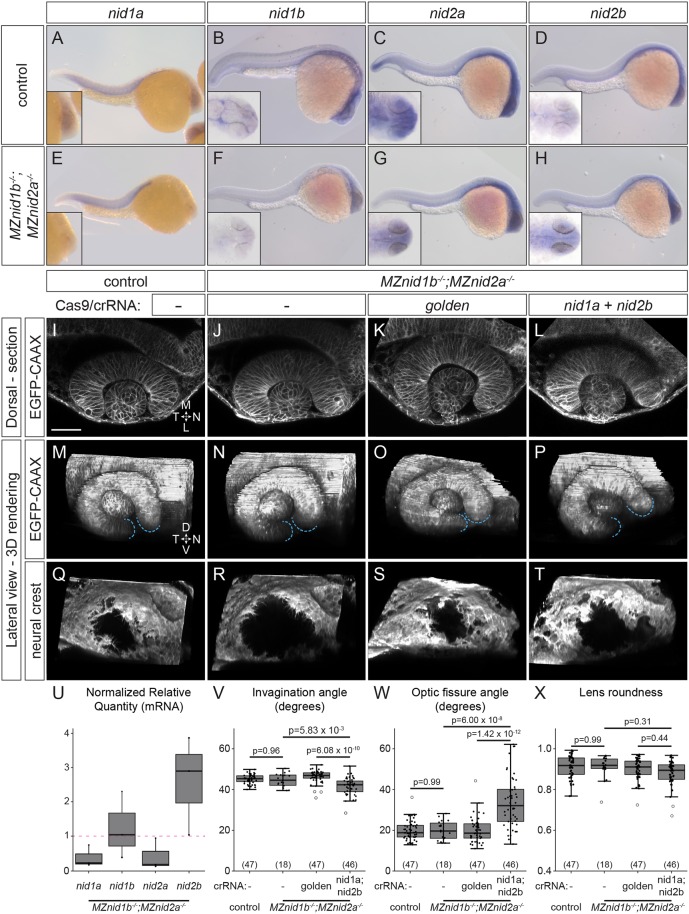
**Nidogen is required for optic cup morphogenesis.** (A-H) Whole-mount *in situ* hybridization for nidogens in 24 hpf control (A-D) and *MZnid1b; MZnid2a* mutant (E-H) embryos. Insets show magnified view of head. (I-T) Optic cup morphology and neural crest localization in EGFP-CAAX mRNA-injected, *Tg(sox10:memRFP)^+^* embryos: control (I,M,Q), *MZnid1b;MZnid2a* mutant (J,N,R), *golden* crispant *MZnid1b;MZnid2a* mutant (K,O,S) and *nid1a;nid2b* crispant *MZnid1b;MZnid2a* mutant (L,P,T). (U) RT-qPCR quantification of nidogen transcripts in *MZnid1b;MZnid2a* mutant embryos, normalized to wild-type control expression levels (magenta dashed line, NRQ=1). Results from three biological replicates run in triplicate. (V-X) Quantification of invagination angle (V), optic fissure angle (W) and lens roundness (X). *n* (embryos) shown in base of graph. *P*-values were calculated using one-way ANOVA with Tukey HSD post-hoc test. Scale bar: 50 μm. D, dorsal; L, lateral; M, medial; N, nasal; T, temporal; V, ventral.

Although our previous experiments suggested that neural crest-derived nidogen might play a role in optic cup morphogenesis, we sought to determine whether endogenous nidogen is necessary. Because we detected *nid1b* and *nid2a* transcripts in neural crest cells around the developing eye, we assayed *nid1b^sa13713^* splice site and *nid2a^sa15802^* premature stop mutants as single and zygotic double mutants but found no obvious optic cup defects (data not shown). *nid1b;nid2a* double mutants are adult viable and fertile; therefore, we also assayed optic cup morphogenesis in maternal-zygotic (MZ) *nid1b;nid2a* double-mutant embryos. Whole-mount *in situ* hybridization suggested that both genes are downregulated or absent in the cranial neural crest (and other tissues) of *MZnid1b;MZnid2a* mutants compared with control embryos (Fig. 7B,C,F,G), and antibody staining revealed a dramatic reduction in BM-localized nidogen 1 (Fig. S5), confirming the specificity of the antibody. However, to our surprise, *MZnid1b;MZnid2a* mutant eyes were phenotypically indistinguishable from controls at 24 hpf (Fig. 7I,J,M,N,Q,R,V-X). As nidogen proteins can function redundantly in the BM (Salmivirta et al., 2002), compensation by other nidogen genes might phenotypically mask loss of *nid1b* and *nid2a* during optic cup development, as previously observed in mouse and zebrafish nidogen single mutants (Bader et al., 2005; Böse et al., 2006; Zhu et al., 2017). In certain mutants, compensatory upregulation of closely related transcripts may be a common phenomenon (El-Brolosy et al., 2019). Using *in situ* hybridization and RT-qPCR (Fig. 7A-H,U), we observed that expression of other nidogens is altered in *MZnid1b;MZnid2a* mutants: *nid2b* transcripts were upregulated to nearly three times the wild-type levels in *MZnid1b;MZnid2a* mutants, whereas, somewhat surprisingly, *nid1a* mRNA levels were decreased. Thus, we hypothesize that compensatory upregulation of *nid2b* could be sufficient to support optic cup morphogenesis in the absence of *nid1b* and *nid2a*.

To test this, we used CRISPR/Cas9 mutagenesis to generate transient *nid1a;nid2b* mutants (crispants) in the *MZnid1b;MZnid2a* background, for a putative quadruple mutant. We targeted both *nid1a* and *nid2b* to avoid compensation by any functional nidogen genes, and targeted *golden* (*slc24a5*) as an independent control locus (Fig. 7I-T; Hoshijima et al., 2016). We found that nidogen crispants phenocopy *tfap2a;foxd3* double mutants (Fig. 7L,P,T): invagination was impaired in nidogen crispants (41.6±1.2°) compared with uninjected (44.6±1.5°) or *golden* crispant controls (46.3±0.9°; Fig. 7V), and optic fissure formation was disrupted in nidogen crispants (33.7±3.7°) versus uninjected (19.9±2.1°) or *golden* crispant controls (20.1±1.8°; Fig. 7W). Nidogen does not appear to be necessary for neural crest migration or survival, as *sox10:memRFP*^+^ cells still surround the RPE in nidogen crispants (Fig. 7T), as in uninjected or *golden* crispant controls (Fig. 7R,S), suggesting that loss of nidogen directly affects optic cup morphogenesis, rather than being a secondary effect due to impairing neural crest. In contrast to the effects of DI-Nid1a overexpression, *nidogen* crispants did not display lens morphogenesis defects at 24 hpf (Fig. 7L,X), suggesting that genetic disruption of all nidogens impairs ECM assembly and morphogenesis differently than DI-Nid1a. Together, these results demonstrate that nidogen is a crucial component of the ECM required to support multiple aspects of optic cup morphogenesis, and that loss of nidogens phenocopies loss of neural crest.

## DISCUSSION

**Fig. 8.**
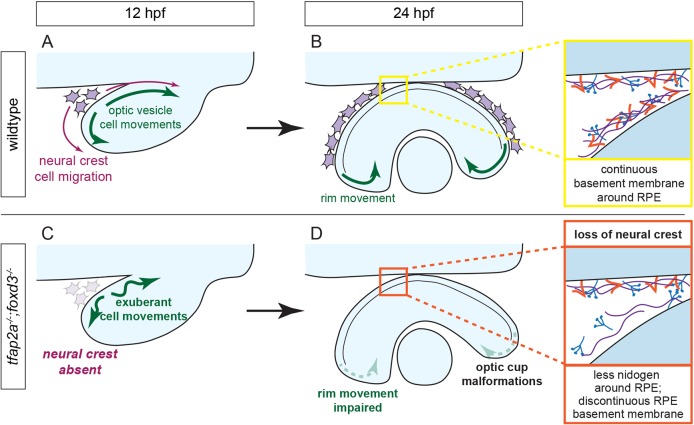
**Model of optic cup morphogenesis in wild-type and *tfap2a;foxd3* double-mutant zebrafish.** (A,B) Optic cup morphogenesis in a wild-type embryo. Neural crest cells migrate around the optic vesicle and enable efficient movement of optic vesicle cells (A). Cells undergo rim movement and contribute to the neural retina, partially enabled by the presence of a continuous BM along the surface of the RPE (B). (C,D) Optic cup morphogenesis in a *tfap2a;foxd3* double-mutant embryo. Most neural crest cells are absent, resulting in optic vesicle cells that move faster and farther than those in wild-type embryos (C). Rim movement is impaired in the absence of a complete, continuous BM around the RPE, resulting in optic cup malformations (D).

Here, we demonstrate that early eye morphogenesis requires neural crest, and present a working model in Fig. 8. The zebrafish *tfap2a;foxd3* double mutant, in which neural crest is genetically disrupted (Arduini et al., 2009; Wang et al., 2011), exhibits aberrant invagination cell movements and discontinuous, disrupted basement membranes, specifically around the developing RPE. We identify the matrix-bridging protein nidogen as a key component of the periocular ECM that is provided in part by neural crest; disruptions to nidogen function impair optic cup morphogenesis.

### Mesenchymal regulation of epithelial morphogenesis

Developing epithelia of the lung, kidney, salivary gland and tooth require mesenchymal interactions to acquire their mature, functional structures (Bader et al., 2005; Ekblom et al., 1994; Kadoya et al., 1997; Thesleff, 2003). Prior work has implicated mesenchyme in eye development: disruptions to POM cause profound optic cup morphogenetic defects (Bassett et al., 2010; Lupo et al., 2011). Although POM has long been observed in close proximity to the developing optic cup, the POM is a heterogeneous mix of neural crest and mesodermally derived mesenchymal cells (Gage et al., 2005; Johnston et al., 1979), and specific molecular contributions from either tissue during early eye development are not well understood.

Several signaling pathways crucial for eye development are regulated in part by POM, including Hh, TGFβ and Wnt (Fuhrmann et al., 2000; Grocott et al., 2011; Sedykh et al., 2017), thus neural crest cells could drive optic cup morphogenesis by modulating morphogen signaling. However, in zebrafish *tfap2a;foxd3* mutants, Pax2a expression is not expanded in a manner consistent with aberrant Hh signaling (Gordon et al., 2018; Lee et al., 2008; Sedykh et al., 2017), nor is TGFβ signaling altered. The remaining mesodermal mesenchyme may be sufficient for proper Hh and TGFβ signaling in zebrafish; additional studies will be necessary to dissect specific roles of mesodermal mesenchyme during optic cup morphogenesis.

### Eye morphogenesis requires neural crest-mediated ECM assembly

The periocular ECM plays key roles in optic cup morphogenesis. In retinal organoid culture, the exogenous components (beyond media) required to elicit optic cup morphogenesis are nodal (a TGFβ ligand) and the ECM components laminin-1 and nidogen (Eiraku et al., 2011). Laminin is required for cell survival, apicobasal polarity and cell movements (Bryan et al., 2016; Ivanovitch et al., 2013; Nicolás-Pérez et al., 2016; Sidhaye and Norden, 2017), and fibronectin is crucial for lens formation (Hayes et al., 2012; Huang et al., 2011). Individual ECM components may each regulate specific, non-overlapping aspects of eye morphogenesis.

The dynamics of ECM deposition and assembly may be crucial for regulating cell movements. Using electron microscopy, we uncovered a loss of BM integrity specifically around the RPE in the *tfap2a;foxd3* double mutant, although major ECM components such as laminin and fibronectin are still present. Comparison of this phenotype to the *laminin-α1* mutant reinforces the functional specificity of BM molecules: loss of laminin leads to dramatically disrupted tissue polarity and cell death in the prospective RPE (Bryan et al., 2016), yet loss of RPE BM does not. Instead, the assembled BM may serve as a scaffold that enables efficient cell movements and RPE flattening, or as a dynamic substrate that provides mechanical signaling. The Hippo signaling pathway is a major cellular mechanotransducer that can respond to ECM stiffness (Chakraborty et al., 2017), and RPE development requires Yap and Taz, transcriptional co-activators of the Hippo pathway (Miesfeld et al., 2015). Although RPE differentiation appears to occur normally without assembled BM in the *tfap2a;foxd3* mutant (Fig. S1O-P), it is an intriguing possibility that Hippo signaling could serve as a molecular link between the BM and morphogenetic movements.

Nidogens are widespread components of BMs (Ho et al., 2008) and although epithelial morphogenesis of the lung and kidney depend on nidogen, the surrounding mesenchyme is those tissues' sole source of nidogens (Bader et al., 2005; Ekblom et al., 1994; Kadoya et al., 1997; Senior et al., 1996). Here, we find that neural crest cells around the RPE produce nidogen; preventing nidogen production through loss of neural crest cells or disrupting nidogen function via expression of a dominant-interfering form of Nid1a or CRISPR/Cas9 mutagenesis impairs optic cup morphogenesis.

It has been reported that loss of the nidogen-binding site in mouse laminin γ1 results in a higher penetrance of renal defects than loss of both nidogen 1 and 2 (Bader et al., 2005; Willem et al., 2002). This was thought to be due to an increase in free nidogen (not bound to laminin), which could act as a dominant-negative factor and interfere with proper matrix assembly. This may explain why ubiquitous overexpression of full-length Nid1a also causes slight defects in optic cup morphogenesis, and suggests that an optimal level of nidogen is required for proper BM architecture. Although nidogen is primarily thought to crosslink laminin with collagen IV, it can also bind other components of the BM, such as heparan sulfate proteoglycans and fibulin (Kalluri, 2003; Lössl et al., 2014; Pozzi et al., 2017); we do not yet fully grasp the molecular underpinnings of BM disruption when nidogen function is impaired. Moving forward, studies of other nidogen binding partners in eye development will be crucial.

Both laminin and fibronectin are still found around the RPE in the absence of neural crest cells, but BM integrity at this site is significantly disrupted. Proper ECM interactions regulate many aspects of eye development, including rim movement (Bryan et al., 2016; Martinez-Morales et al., 2009; Sidhaye and Norden, 2017), a process we show to be disrupted in the absence of neural crest. We propose that through deposition of nidogen, and possibly other factors, neural crest cells contribute to a specific microenvironment, which directs the proper movements of optic vesicle cells. In this model, the BM surrounding the RPE serves as a handbrake, restricting cell movements within the optic vesicle to ensure their correct speed and migratory course during optic cup morphogenesis. It will be interesting to investigate exactly what aspect of assembled BM function is required to support these specific morphogenetic events: future studies will address signaling, structural and mechanical aspects of ECM assembly and activity.

## MATERIALS AND METHODS

### Zebrafish lines

Embryos from the following zebrafish (*Danio rerio*) mutant and transgenic lines were collected within 10 min of fertilization, raised at 28.5°C and staged according to hours post fertilization and morphology (Kimmel et al., 1995). Specifically, embryos were staged as 12 hpf by counting six somites, and as 24 hpf based on pigment in the developing RPE becoming visible.

#### Mutant alleles

For details of *tfap2a^ts213^;foxd3^zdf10^*, see Arduini et al. (2009). *nid1b^sa13713^* contains a splice site mutation, and *nid2a^sa15802^* contains a premature stop mutation (Kettleborough et al., 2013). The *alyron^z24^* allele contains a C-to-A transversion in the coding sequence of the *paf1* gene, resulting in a premature stop mutation at tyrosine 281 (Y281*) (Jurynec et al., 2019).

#### Transgenic alleles

Transgenic alleles used were: *Tg(sox10:memRFP)^vu234^* (Kirby et al., 2006), *Tg(sox10:GFP)^ba4^* (Dutton et al., 2008), *Tg(bactin2:EGFP-CAAX)^z200^* (Gordon et al., 2018), *Tg(hsp70:lyn-mCherry-2A-WT-Nid1a)^z202^* and *Tg(hsp70:lyn-mCherry-2A-DI-Nid1a)^z203^*.

### Construction of Nid1a transgenic lines

*Tg(hsp70:lyn-mCherry-2A-WT-Nid1a)^z202^* (also referred to as *hs:WT-Nid1a*) and *Tg(hsp70:lyn-mCherry-2A-DI-Nid1a)^z203^* (also referred to as *hs:DI-Nid1a*) were generated using Gateway (Invitrogen) recombination. IMAGE Clone ID 8000296 (GE Dharmacon) was used as the template to PCR amplify cDNAs encoding wild-type and dominant-interfering Nid1a; these were ligated into pCS2FA prior to Gateway cloning. PCR primers were used to introduce the PTV-2A peptide (Provost et al., 2007) on the 5′ end, and the SV40 late poly-adenylation signal on the 3′ end of the zebrafish *nid1a* cDNA. Gateway 3′ entry clones were generated via BP recombination and subsequently LR recombined into the pDEST-Tol2-CG2 destination vector, which contains a *myl7:EGFP* expression cassette as a transgenesis marker (Kwan et al., 2007). Plasmid DNA (25 pg) was microinjected along with 50 pg mRNA encoding Tol2 transposase into single-cell wild-type embryos, which were screened for *myl7:EGFP* expression. Fluorescent embryos were raised to adulthood and outcrossed to generate stable transgenic lines.

### Heat shocks

Embryos were transferred from a 28.5°C incubator and immediately overlaid with fresh, preheated 39°C E3. Embryos were incubated at 39°C for 1 h on an Echotherm heating plate (Torrey Pines Scientific). Embryos were then transferred back to a 28.5°C incubator and grown to the indicated stage.

### Allele identification/genotyping

All mutant alleles were PCR genotyped using either CAPS or dCAPS techniques (Neff et al., 1998). *tfap2a^ts213^*: Forward (5′-CGCTCAGGTCTTATAAATAGGCTACTAATAATGTTAC-3′), Reverse (5′-CTGAGAGGTGGCTATTTCCCGTTAAGATTCG-3′), mutant allele was cut with BlpI; *foxd3^zdf10^*: dCAPS Forward (5′-CGACTGCTTCGTCAAGATCCCACGGGAACCGGGCAACCCGGGCAAAGGCAACTACTGGACCCTCGACCCCCAGTCGGAAAATAT-3′), Reverse (5′-CAGGGGGAATGTACGGGTACTGC-3′), mutant allele was cut with SspI; *paf1^z24^*: Forward (5′-GTTCAGAGGTATGATGGATGAGG-3′), Reverse (5′-GTATGCAGCTTTATGAAAACACTC-3′), wild-type band was cut with NspI; *nid1b^sa13713^:* Forward (5′-ATCTGGGCAGTCCTGAAGGAATCGCC-3′), dCAPS Reverse (5′-GCACATTCTGGAGCTCATTCTGATTCTGATTTTAAACGTTCGCGCTGCTCTACTTTAGCGTGTTTTAGCCGTGTCATGCATTGGT-3′), wild-type allele was cut with KpnI; *nid2a^15802^:* Forward (5′-GACTTGCATTTCCAGTTACTCAGAATAAATATCTGTCTAGAC-3′), dCAPS Reverse (5′-CGGCCGTTGCCGTAAAAGCCGGAGTTGCAGTGACAGCAGAAGCCGGTGGAATGATCTGTACAGTGAGCGTTTAGACTGCAGT-3′), mutant allele was cut by ScaI.

### RNA synthesis and injections

Capped mRNAs were synthesized using linearized pCS2 templates (pCS2-EGFP-CAAX, pCS2FA-H2A.F/Z-mCherry), the mMessage mMachine SP6 kit (AM1340, Invitrogen), and purified using the Qiagen RNeasy Mini Kit and ethanol precipitated. Each mRNA (150-250 pg) was microinjected into the cell of one-cell-stage embryos. EGFP-CAAX mRNA was injected to visualize cell membranes, H2A.F/Z-mCherry mRNA was injected to visualize nuclei.

### Antibody staining

Embryos for most antibodies were fixed at the indicated stage in 4% paraformaldehyde, permeabilized in PBST (PBS+0.5% Triton X-100), and blocked in PBST+2% bovine serum albumin. Antibodies and concentrations were as follows: anti-Pax2a (GTX128127, Genetex; 1:200); anti-pSmad3 (ab52903, Abcam; 1:200); anti-Laminin 1 (L9393, Sigma-Aldrich; 1:100); anti-fibronectin (F3648, Sigma-Aldrich; 1:100); anti-GFP (A10262, Invitrogen; 1:200).

In accordance with methods described by Carrara et al. (2019), anti-Nidogen 1/Entactin (ab14511, Abcam) was used at 1:100 and required a modified embryo preparation after 4% paraformaldehyde fixation: embryos were permeabilized in 30 μg/ml Proteinase K for 15 min, and blocked in 0.8% PBST+10% sheep serum+1% bovine serum albumin. The antibody was applied in 0.8% PBST+1% sheep serum+1% bovine serum albumin.

Secondary antibodies used were: Alexa Fluor 488 goat anti-mouse (A-11001, Invitrogen), Alexa Fluor 488 goat anti-rabbit (A-11008, Invitrogen), Alexa Fluor 488 goat anti-chicken (A-11039, Invitrogen), all used at 1:200. Nuclei were detected by incubation with 1 µM TO-PRO-3 iodide (T3605, Invitrogen). Embryos were cleared through a series of 30%/50%/70% glycerol (in PBS) prior to imaging.

### *In situ* hybridization

Embryos were fixed at the indicated stage in 4% paraformaldehyde overnight at 4°C and dehydrated in 100% methanol. Color *in situ* hybridizations were performed as described previously (Thisse and Thisse, 2008). Fluorescent *in situ* hybridizations were carried out as described previously (Lauter et al., 2014; Leerberg et al., 2017). Anti-GFP labeling and detection was performed after *in situ* hybridization and tyramide signal amplification.

*In situ* probes were synthesized from linearized pBluescript II SK+ templates (pBSII-Nid1a, pBSII-Nid1b, pBSII-Nid2a, pBSII-Nid2b) using T3 or T7 polymerases and DIG labeling mix (11277073910, Roche). All four probe sequences were synthesized (IDT gBlocks, IDT) and ligated into pBluescript II SK+.

### Light microscopy

For time-lapse imaging, 12 hpf embryos were embedded in 1.6% low-melt agarose (in E3) in DeltaT dishes (Bioptechs, 0420041500 C); E3 was overlaid and the dish covered to prevent evaporation. For antibody staining or fluorescent *in situ* hybridization imaging, embryos were embedded in 1% low-melt agarose (in PBS) in Pelco glass-bottom dishes (14027, Ted Pella); PBS was overlaid to prevent evaporation.

Confocal images were acquired using a Zeiss LSM710 laser-scanning confocal microscope. For time-lapse imaging, datasets were acquired using the following parameters: 63 *z*-sections, 2.10 µm *z*-step, 40× water-immersion objective (1.2 NA). Time between *z*-stacks was 3 min 30 s (Movies 1 and 2), 2 min 45 s (Movies 4, 6, 8 and 10) and 2 min 30 s (Movies 5, 7, 9 and 11). Movies 4-11 are average projections of the EGFP-CAAX channel (gray) through ∼60 µm centered at the dorsal/ventral midpoint of the optic vesicle with indicated nuclear trajectories overlaid. For all time-lapse and antibody imaging, datasets were acquired without knowledge of embryo genotype. Embryos were de-embedded and genotyped after imaging was completed. Movies 5, 7, 9 and 11 extend beyond 24 hpf, but all cell trajectory analyses were performed only up to the 24 hpf time point.

Brightfield images were acquired using an Olympus SZX16 stereomicroscope with an Olympus DP26 or UC90 camera.

### TEM

At 24 hpf, embryos were fixed, stained and embedded using the microwave-assisted tissue processing protocol described by Czopka and Lyons (2011). Tails were dissected from embryos prior to fixation and used for genotyping.

Our tissue sampling and analytical techniques have been described previously in detail (Anderson et al., 2011a,b; Lauritzen et al., 2013; Marc et al., 2013, 2014).

The tissues were osmicated for 60 min in 0.5% OsO_4_ in 0.1 M cacodylate buffer, processed in maleate buffer for *en bloc* staining with uranyl acetate, and processed for resin embedding. The epoxy resin bloc with zebrafish tissue was sectioned in the horizontal plane at 70-90 nm onto polyvinyl formal resin-coated copper slot grids for TEM (Lauritzen et al., 2013; Marc and Jones, 2002).

Each TEM section was imaged on a JEOL JEM-1400 transmission electron microscope at 5000× or 20,000×; images were stored in 16- and 8-bit versions, as well as image pyramids of optimized tiles for web visualization with the Viking viewer (Anderson et al., 2011a,b). Each image was captured as an array of image tiles at 330-465 tiles/slice (5000×) or 60-998 tiles/slice (20,000×) with 15% overlap.

### Image processing and analysis

Images were processed using Fiji (Schindelin et al., 2012). Volume rendering was performed using FluoRender (Wan et al., 2009, 2017). For lateral view 3D rendering of the optic cup, the ectoderm was digitally erased in ImageJ prior to visualization in FluoRender. Invagination angles were measured as previously described (Bryan et al., 2016) and as illustrated in Fig. 1G; optic fissure angles were measured as previously described (Gordon et al., 2018) and as illustrated in Fig. 1H. Pax2a- and pSmad3-positive regions were measured as angles (see Fig. S2L-M) by setting the vertex at the center of the lens (Pax2a) or the innermost point of retinal invagination (pSmad3). The pSmad3^+^ portion of the lens was measured at the lens dorsal/ventral midpoint and calculated by dividing the area of pSmad3 staining by the total lens area. Lens roundness was measured by drawing a region of interest around the lens at its dorsal/ventral midpoint, followed by analysis using the Fiji Shape Descriptors measurement tool (‘Roundness’). Nidogen coverage around the optic cup was measured at the dorsal/ventral midpoint of the optic cup by tracing the length of the nidogen-positive surface around each specific tissue of interest (brain, RPE or lens) and dividing that by the total length of that tissue. For the brain, this entailed measuring from the posterior limit of the image to the posterior margin of the olfactory placode; the entire RPE was measured starting where the temporal and nasal sides of that tissue contact the surface ectoderm; the entire circumference of the lens was measured. A segment was considered ‘nidogen negative’ if it was a clearly defined basal surface that lacked staining, adjacent to a nidogen-positive basal surface, and the signal-deficient region also lacked signal in neighboring sections in the *z*-stack. Laminin-1 and fibronectin coverage were measured in a similar fashion, but all optic tissues were binned into one surface around the optic cup. For all relevant measurements, the lens dorsal/ventral midpoint was derived independently for each optic cup from 3D image volume data, and validated via 3D rendering and visualization in FluoRender. BM lengths were measured using Viking: basal lamina coverage was considered continuous where a discernable lamina lucida and lamina densa were both visible lining the plasma membrane; otherwise that region was considered a discontinuous gap in coverage (Feitosa et al., 2011; Yurchenco and Schittny, 1990). Continuity percentages were calculated by dividing the length of continuous BM visible along a tissue by the total length of the tissue surface. For Fig. 5, *sox10:GFP*^+^ cells were counted as near the optic fissure if they were located in the optic fissure or were in contact with a margin of the optic fissure. Individual cell tracking was performed as described by Kwan et al. (2012) using LongTracker; nuclei were visualized using H2A.F/Z-mCherry.

### Statistical analysis

*P*-values for comparisons between two groups were calculated using a two-tailed Welch's *t*-test; Welch's *t*-test was chosen as it accounts for unequal sample sizes and inherently accounts for the possibility of unequal variance between groups. One-way ANOVA with a Tukey HSD post-hoc test was used when comparing multiple groups. Statistical significance was assumed using a confidence interval of 95%. Box and whisker plots were generated using the ggplot2 package in RStudio. The upper and lower ‘hinges’ correspond to the first and third quartiles. The upper whisker extends from the upper hinge to the highest value within (1.5×IQR), where IQR is the inter-quartile range. The lower whisker extends from the lower hinge to the lowest value within (1.5×IQR). Data points outside of the ends of the whiskers (depicted as empty, white circles) are outliers.

### Real-time quantitative PCR

Embryos were pooled at 24 hpf (*n*=30) and either flash-frozen in liquid nitrogen and stored at −80°C until use, or immediately homogenized using the QIAshredder (79654, Qiagen). Total RNA was then extracted using the RNeasy Mini Kit (74104, Qiagen) and stored at −80°C until use. cDNA was synthesized using the iScript cDNA Synthesis kit (1708890, Bio-Rad) following the manufacturer's recommendations, such that 1 µg of RNA was loaded into each reaction. Three biological replicates were collected for each condition.

RT-qPCR primers were designed to span more terminal exon-exon junctions and produce amplicons of ∼100 bp in length (Table S1). During optimization, products were both gel analyzed and sequenced to ensure product specificity. All reactions utilized the PowerUp SYBR Green Master Mix (A25741, Applied BioSystems) and were performed on an Applied BioSystems 7900HT instrument. Efficiency (E) was calculated using a four-point standard curve and optimized to be ∼2.0 (Carleton, 2012). Wild-type 24 hpf cDNA was used to calculate efficiencies for *nid1a*, *nid1b* and *nid2a*, whereas a 1:1 mixture of 24 hpf wild-type and MZ*nid1b^−/−^;*MZ*nid2a^−/−^* cDNA was used to calculate the efficiency for *nid2b*. Cycling parameters were: 50°C (2 min) followed by 40 cycles of 95°C (2 min), 58°C (15 s), 72°C (1 min), then followed by a dissociation curve. Applied BioSystems software SDSv2.4 was used to determine cycle threshold (C_t_) values and melting curves. All reactions were performed in triplicate with a ‘no-template’ control.

RT-qPCR analysis was performed in Microsoft Excel using the ΔΔCt method (Livak and Schmittgen, 2001; Vandesompele et al., 2002). The relative quantity (RQ) of each *nid* gene was normalized to the reference gene *eef1a1l1* (Karra et al., 2015), and the normalized relative quantity (NRQ) was determined by normalizing 24 hpf *MZnid1b^−/−^;MZnid2a^−/−^* expression to normalized 24 hpf wild-type expression (Fig. 7I).

### CRISPR-Cas9

Mutagenesis was achieved using the Alt-R CRISPR-Cas9 system developed by IDT. CRISPR RNAs (crRNAs) were designed to target exon 3 of *nid1a* (5′-UCGCAACACAGAGGACACAGAGG-3′) and exon 5 of *nid2b* (5′-GGACCUCCAUGGCUUUGCGGUGG-3′), or against a site within intron 5 of *slc24a5* (*golden*) (5′-AUAAAGUGAGGAGUGAUGGG-3′), published by Hoshijima et al. (2016). The crRNA and trans-activating crRNA (tracrRNA) were annealed into a dual guide (dg)RNA complex at a 1:1 ratio and stored at −20°C until use. On the day of injections, the ribonucleoprotein was assembled by incubating the dgRNA complexes (25 µM of total dgRNA) with the Cas9 Nuclease 3NLS (25 µM) (1074181, IDT) at 37°C for 5 min (Hoshijima et al., 2019). To visualize eye morphogenesis in crispants, as well as to determine which embryos had been successfully injected, 150 pg of capped EGFP-CAAX mRNA was co-injected with the ribonucleoprotein complex. Microinjections were performed into the cell of one-cell-stage embryos.

### Capillary electrophoresis/fragment analysis

PCR primers were designed to generate a ∼80 bp amplicon flanking the crRNA target site of either *nid1a* or *nid2b.* The 5′ primer included a 5′ 6-FAM fluorescent tag (IDT) to enable fragment detection. Genomic DNA was isolated from *MZnid1b^−/−^;MZnid2a^−/−^* 1 day post-fertilization embryos injected with crRNA targeting the *nid1a* and *nid2b* loci or the *slc24a5* (*golden*) locus. Capillary electrophoresis was performed on an Applied BioSystems 3730 instrument.

Analysis was adapted from the method of Carrington et al. (2015). Fragments below 40 bp were removed from the analysis, as were fragments with a signal height below 1000. Six control, *golden-*injected embryos were analyzed for each mutagenesis experiment, and fragments above the specified thresholds were considered ‘WT’ if detected in at least two control embryos. Fragments observed in *nid1a/nid2b*-injected embryos were identified as being ‘WT’ if they were within ±0.5 bp length of fragments observed in control embryos, or were otherwise assigned as ‘Mutant’ if they were of a length unobserved in control embryos. To determine the frequency of edited *nid1a* or *nid2b* alleles in the crRNA *nid1a/nid2b*-injected embryos, the height of either all ‘WT’ peaks or all ‘Mutant’ peaks was divided by total (WT+Mutant) peak height to yield a percentage. Only crispants with a mutant allele frequency >70% for both *nid1a* and *nid2b* were used for further analysis.

## Supplementary Material

Supplementary information
