# Supplementary material for: Optic cup morphogenesis requires neural crest-mediated basement membrane assembly
Source: Development. 2020 Feb 21;147(4):dev181420. doi: 10.1242/dev.181420 (PMC7044464; doi:10.1242/dev.181420)
Supplement: Supplementary information [file develop-147-181420-s1.pdf]

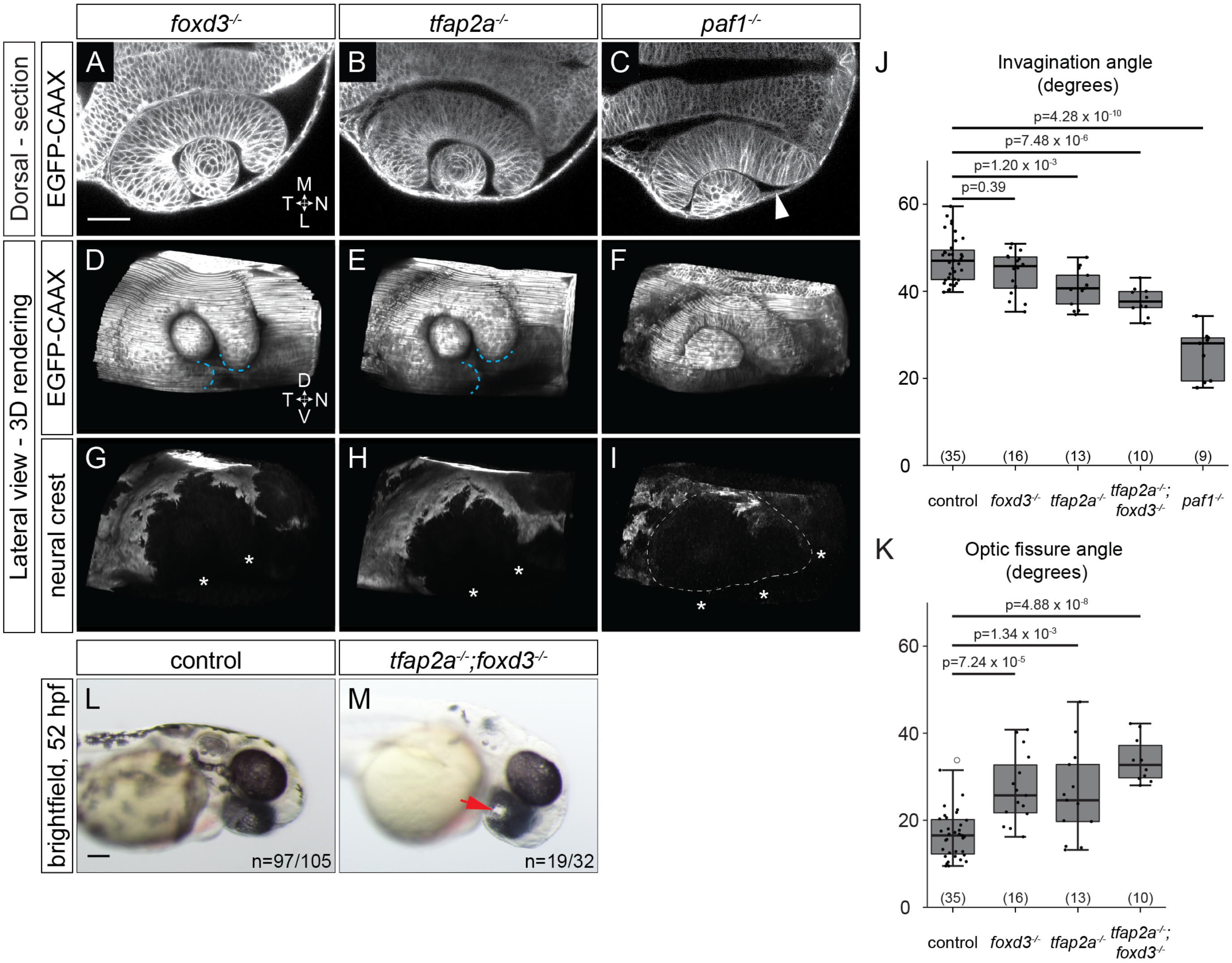

Figure S1. Related to Figure 1. Optic cup morphogenesis defects in neural crest mutants
(A-I) 24 hpf optic cups of *Tg(bactin2:EGFP-CAAX);Tg(sox10:memRFP)* double transgenic *foxd3* (A, D, G), *tfap2a* (B, E, H), and *paf1* (C, F, I) mutant embryos. Single sections shown are at the dorsal/ventral midpoint of the lens; lateral views are a 3D rendering of the corresponding 4D dataset.
(J-K) Quantification of invagination angles (J) and optic fissure angles (K) measured as in Figure 1. Results are from 2-3 experiments; n (embryos) shown at base of each graph. P-values calculated using Welch's t-test, white circles are outliers.
(L-M) Brightfield images of 52 hpf control and *tfap2a;foxd3* mutant embryos. Red arrow in (P) indicates coloboma.
Scale bars: 50 µm (A), 100 µm (L). M, medial; L, lateral; D, dorsal; V, ventral; N, nasal; T, temporal.

Development • Supplementary information

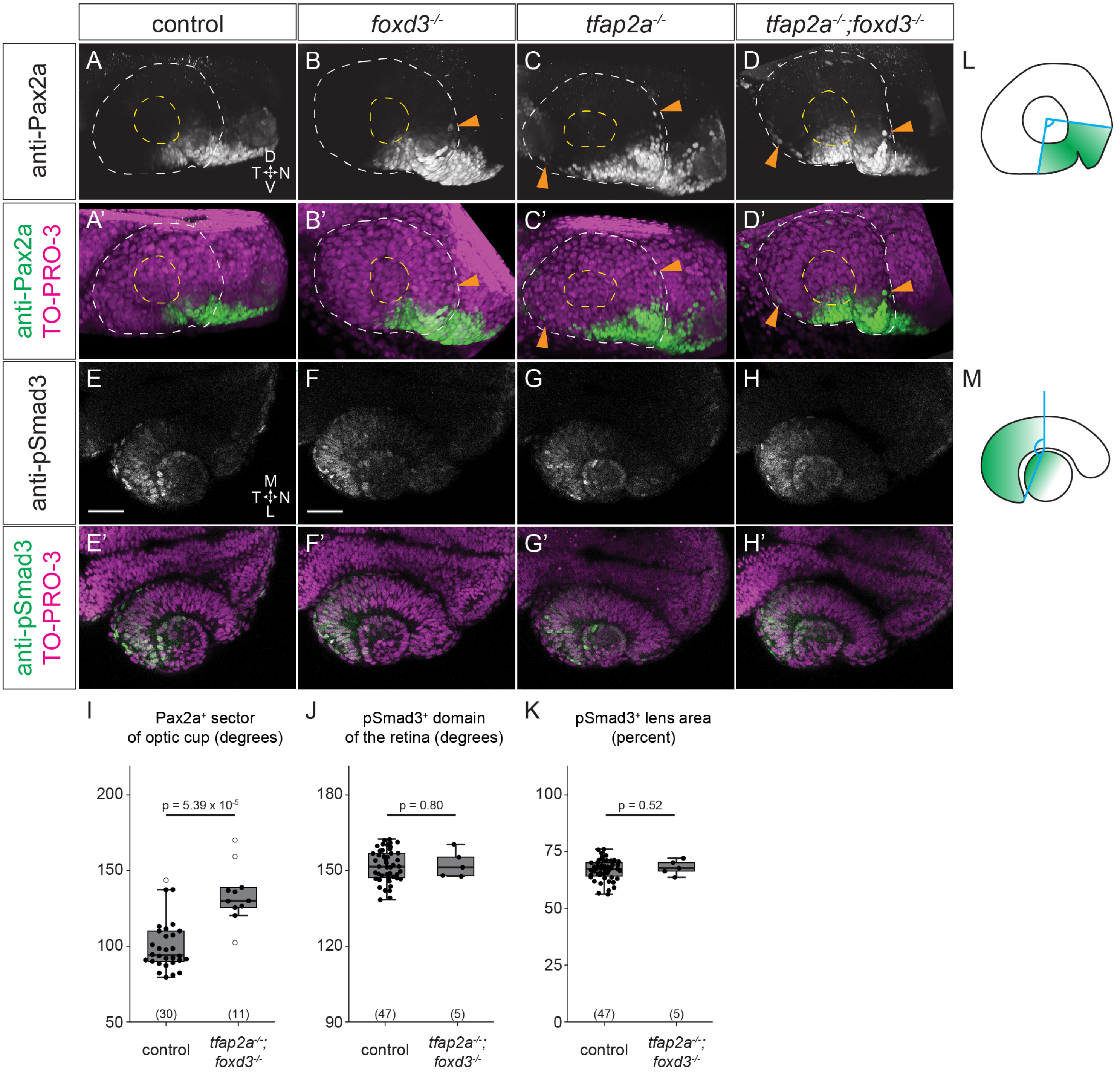
control
foxd3-/-
tfap2a-/-
tfap2a-/-;foxd3-/-
anti-Pax2a
anti-Pax2a
TO-PRO-3
anti-pSmad3
anti-pSmad3
TO-PRO-3
A
B
C
D
L
A'
B'
C'
D'
E
F
G
H
M
E'
F'
G'
H'
D
T N
V
M
T N
L
I
Pax2a+ sector
of optic cup (degrees)
p = 5.39 x 10-5
200
150
100
50
(30)
(11)
control
tfap2a-/-;
foxd3-/-
J
pSmad3+ domain
of the retina (degrees)
p = 0.80
180
150
120
90
(47)
(5)
control
tfap2a-/-;
foxd3-/-
K
pSmad3+ lens area
(percent)
p = 0.52
100
75
50
25
0
(47)
(5)
control
tfap2a-/-;
foxd3-/-

Figure S2. Related to Figure 1. Pax2a is expanded in neural crest mutants while TGF-beta signaling is unaffected

(A-D) Immunofluorescence of Pax2a. Lateral view, 3D renderings of 24 hpf control (A), *foxd3* mutant (B), *tfap2a* mutant (C) and *tfap2a;foxd3* double mutant (D) eyes. White dashed circles denote the boundary of the optic cup, yellow dashed circles display the boundary of the lens. Orange arrowheads in (B-D) indicate RPE cells which ectopically express Pax2a. Immunofluorescence merged with nuclei counterstained with TO-PRO-3 (magenta, A'-D').

(E-H) Immunofluorescence of phospho-Smad3. Dorsal view, single confocal sections of 24 hpf control (E), *foxd3* mutant (F), *tfap2a* mutant (G) and *tfap2a;foxd3* double mutant (H) eyes. Immunofluorescence merged with nuclei counterstained with TO-PRO-3 (magenta, E'-H'). Sections shown are at the dorsal/ventral lens midpoint.

(I-M) Measurements of the Pax2a expressing portion of control and *tfap2a;foxd3* mutant eyes. $Pax2a^{+}$ sectors of the eye were measured from the lateral surface as diagrammed in (L), with the vertex of the angle set at the center of the lens. $pSmad3^{+}$ retinal domain was measured from the lateral margin of the optic cup as diagrammed in (M), with the vertex of the angle set at the innermost point of the central retina. $pSmad3^{+}$ lens area was calculated by dividing the area of the lens expressing pSmad3 over the total lens area. P-values calculated using Welch's t-test, white circles are outliers. Results are from 2-3 experiments; n (embryos) shown in the base of the graph.

Scale bar, 50 µm. M, medial; L, lateral; D, dorsal; V, ventral; N, nasal; T, temporal.

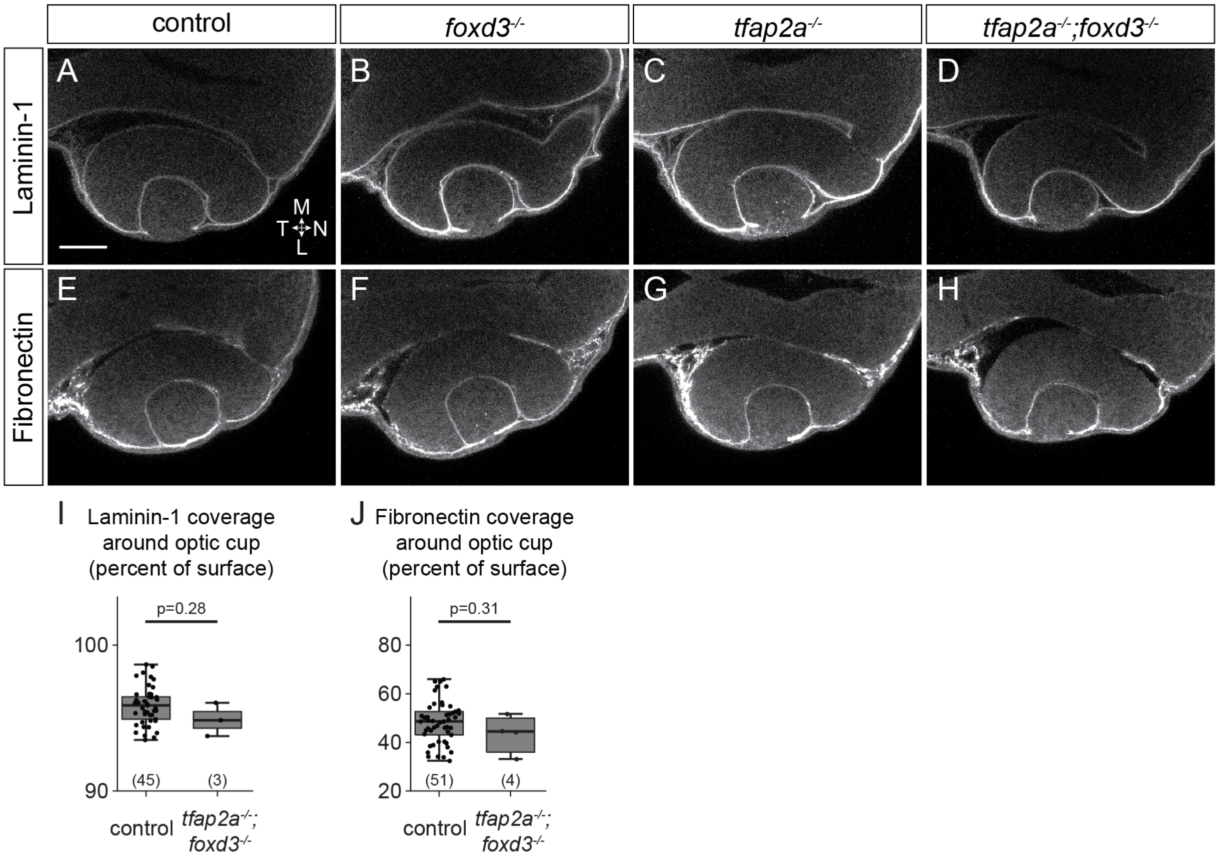

Figure S3. Related to Figure 3. Laminin and fibronectin localization are unaffected in *tfap2a;foxd3* mutants

(A-H) Immunofluorescence for laminin-1 and fibronectin. At 24 hpf, both laminin (A-D) and fibronectin (E-H) are found around the developing optic cup in all genotypes shown.

(I-J) Measurements of laminin-1 (I) and fibronectin (J) distribution around the optic cup. Percentages were calculated by dividing the length of antibody labeling over the total optic cup surface length. P-values calculated using Welch's t-test, results are from 2 experiments each. Scale bar, 50 µm. Dorsal view, single confocal sections. M, medial; L, lateral; N, nasal; T, temporal.

Development • Supplementary information

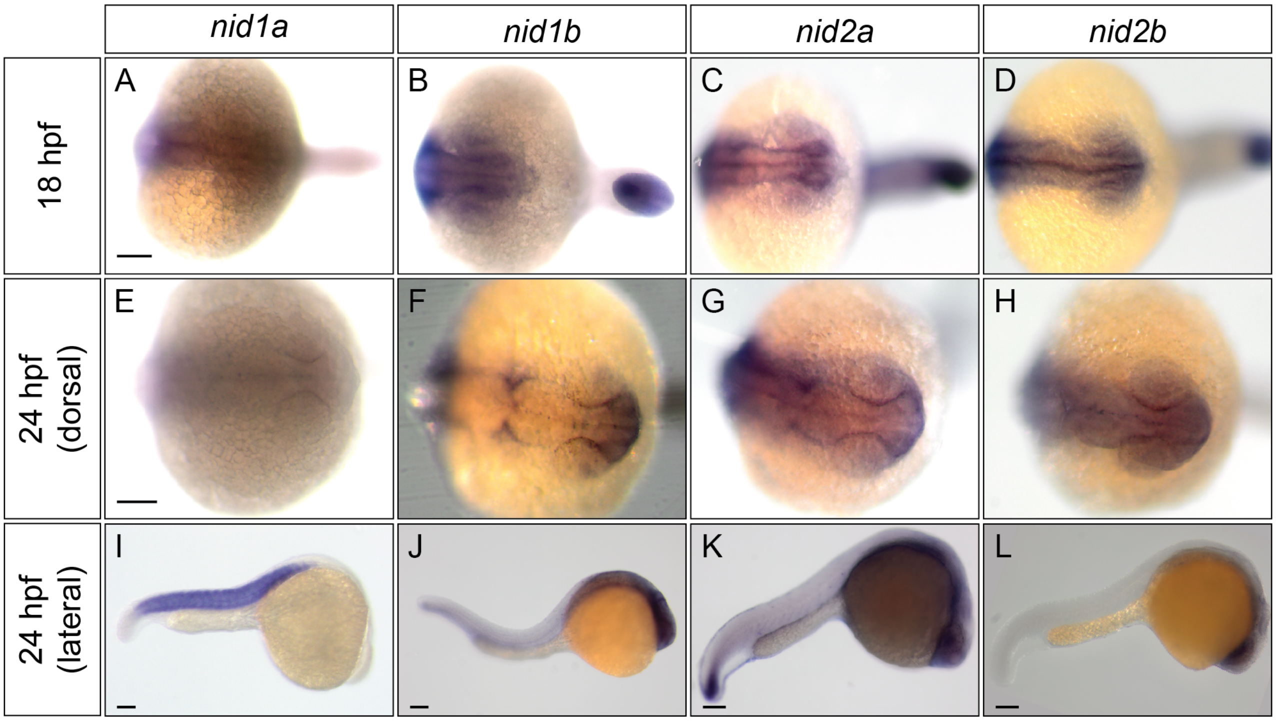

Figure S4. Related to Figure 4. Zebrafish nidogen mRNA expression patterns at 18 and 24 hpf

(A-L) Whole mount *in situ* hybridization with anti-sense probes against indicated *nidogen* mRNA at 18 hpf (A-D) or 24 hpf (E-L). Scale bars, 100 µm.

Development • Supplementary information

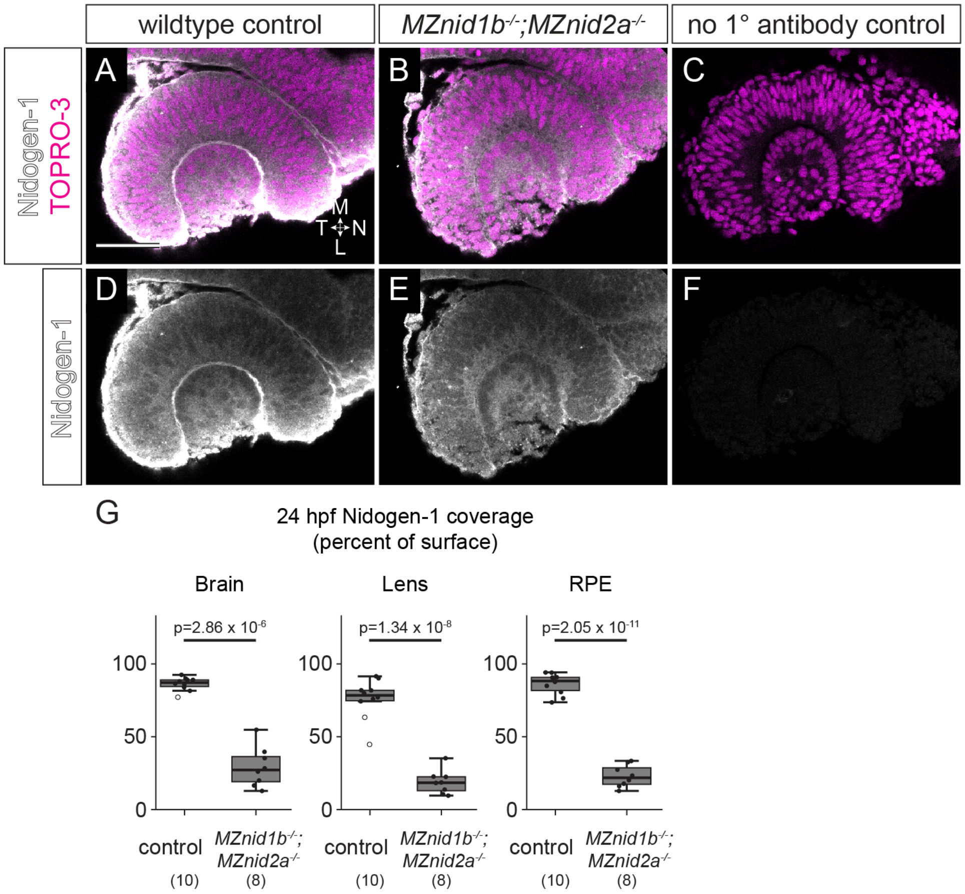

Figure S5. Related to Figure 7. Nidogen-1 protein is reduced in maternal-zygotic *nid1b;nid2a* double mutants

(A-F) Immunofluorescence for nidogen-1. At 24 hpf, nidogen is present in basement membranes throughout the head of wildtype control embryos (A, D), but is severely reduced in the basement membranes of *MZnid1b;MZnid2a* double mutants (B, E). A wildtype embryo which was not exposed to primary antibody is shown as a control in (C, F).

(G) Measurements of nidogen-1 protein coverage around the brain, lens, and RPE. Percentages were calculated by dividing the length of antibody labeling over the total tissue surface length. P-values calculated using Welch's t-test. n (embryos) shown below the corresponding genotype. Scale bar, 50 µm. Dorsal view, single confocal sections. M, medial; L, lateral; N, nasal; T, temporal.

Development • Supplementary information

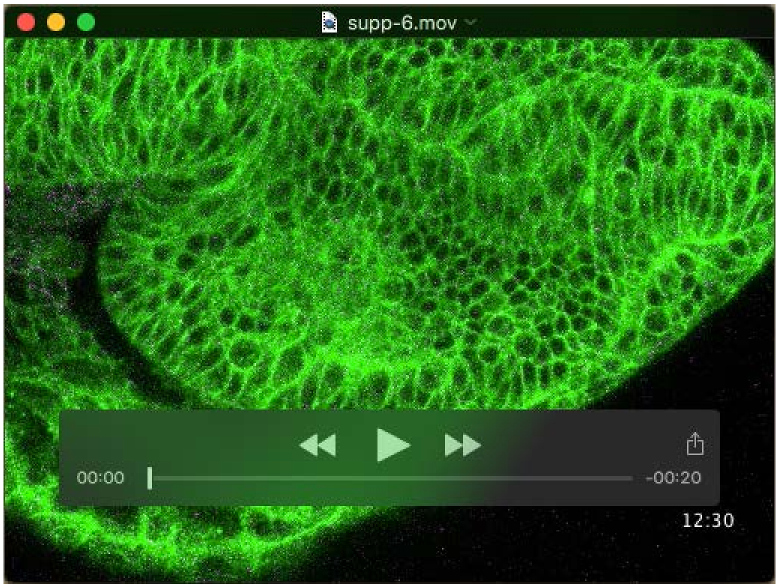

Movie 1. Related to Figure 1. Neural crest migration during optic cup morphogenesis. 12.5-24.5 hpf timelapse of a *Tg(β-actin2:EGFP-CAAX);Tg(sox10:memRFP)* double transgenic embryo. EGFP-CAAX labels all cell membranes (green), while membrane-bound RFP (magenta) labels only the neural crest. Dorsal view, single confocal section through the dorsal/ventral midpoint of the optic cup. Nasal (anterior) is to the right, temporal (posterior) to the left. ΔT between z-stacks, 3 minutes 30 seconds.

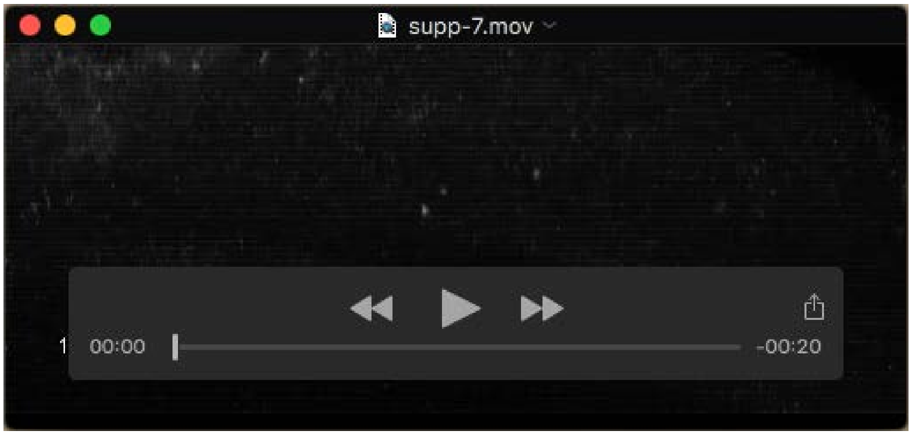

Movie 2. Related to Figure 1. Neural crest migration during optic cup morphogenesis. 12.5-24.5 hpf timelapse of a *Tg(β-actin2:EGFP-CAAX);Tg(sox10:memRFP)* double transgenic embryo. Lateral view, 3D rendering of the same timelapse dataset shown in Movie 1. Only the RFP channel is shown (grayscale) to enable visualization of neural crest cell migration. Nasal (anterior) is to the right, temporal (posterior) to the left, dorsal to the top, ventral to the bottom. ΔT between z-stacks, 3 minutes 30 seconds

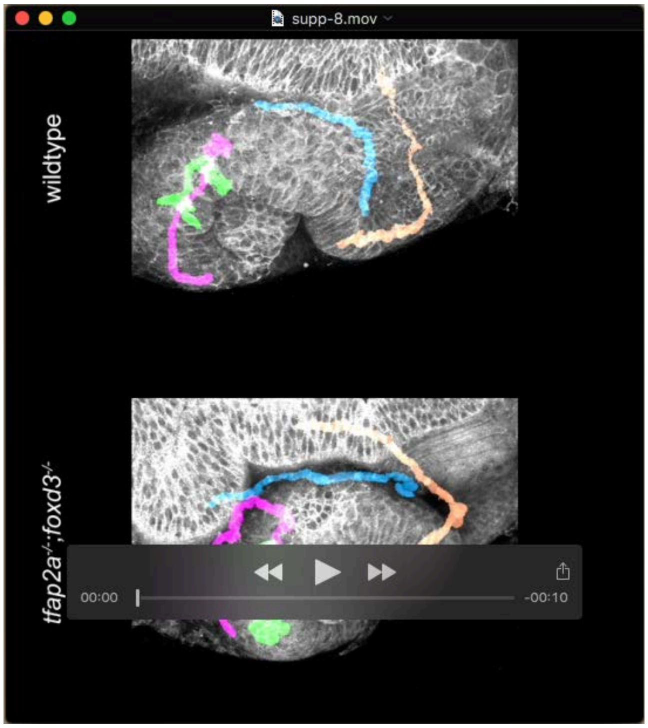

Movie 3. Related to Figure 2. Nuclear trajectories visualized in in three dimensions. 3D rendered rotation of 24 hpf timepoints showing representative 4-dimensional trajectories of nuclei from cells in the nasal retina (orange), nasal RPE (blue), temporal retina (green), and temporal RPE (magenta). Membrane channel is displayed in grayscale.

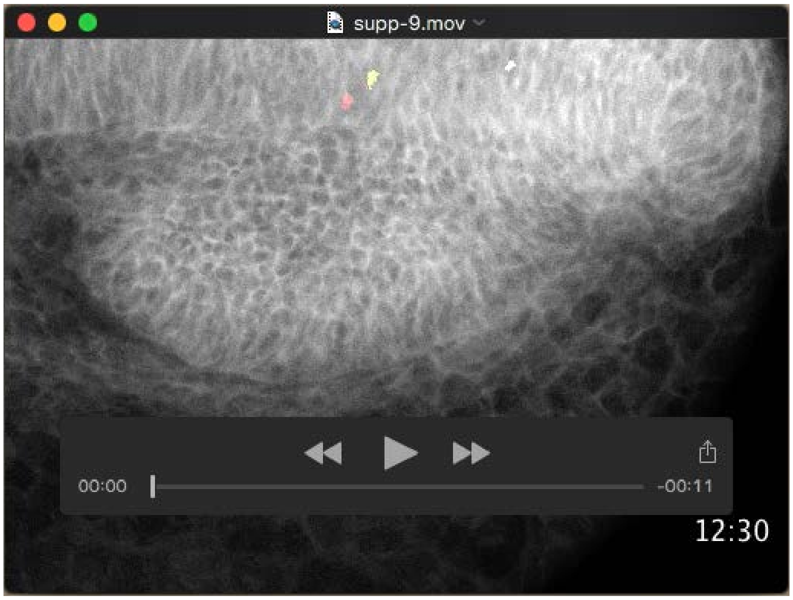

Movie 4. Related to Figure 2. Wildtype nasal retina nuclear trajectories from 12.5-24 hpf. Representative trajectories over membrane channel average (grayscale). ΔT between z-stacks, 2 minutes 45 seconds.

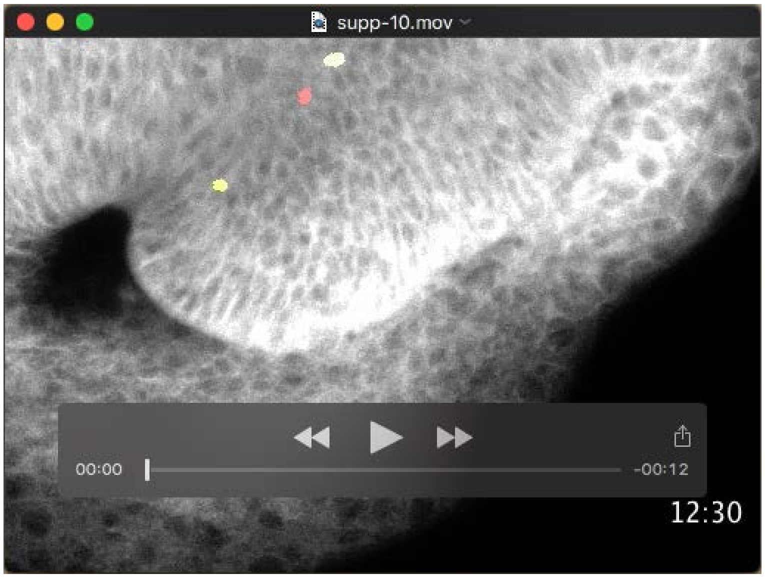

Movie 5. Related to Figure 2. *tfap2a;foxd3* double mutant nasal retina nuclear trajectories from 12.5-24 hpf. Representative trajectories over membrane channel average (grayscale). ΔT between z-stacks, 2 minutes 30 seconds.

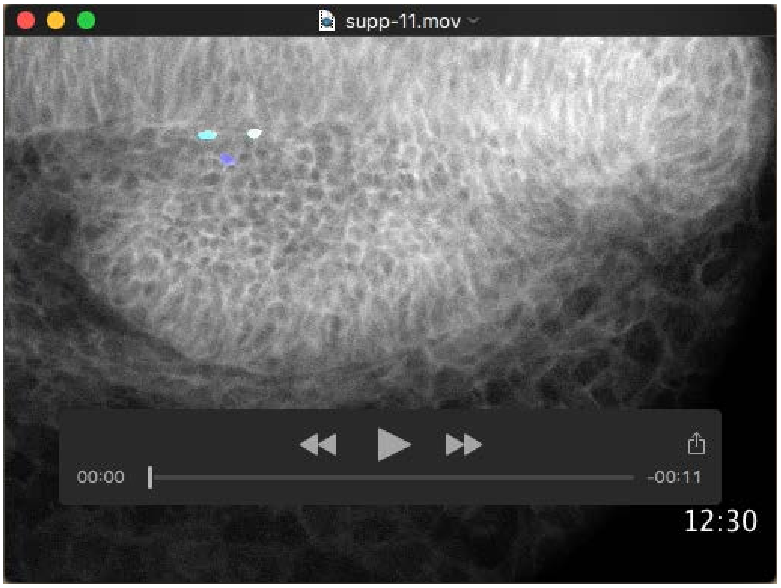

Movie 6. Related to Figure 2. Wildtype nasal RPE nuclear trajectories from 12.5-24 hpf. Representative trajectories over membrane channel average (grayscale). ΔT between z-stacks, 2 minutes 45 seconds.

Development • Supplementary information

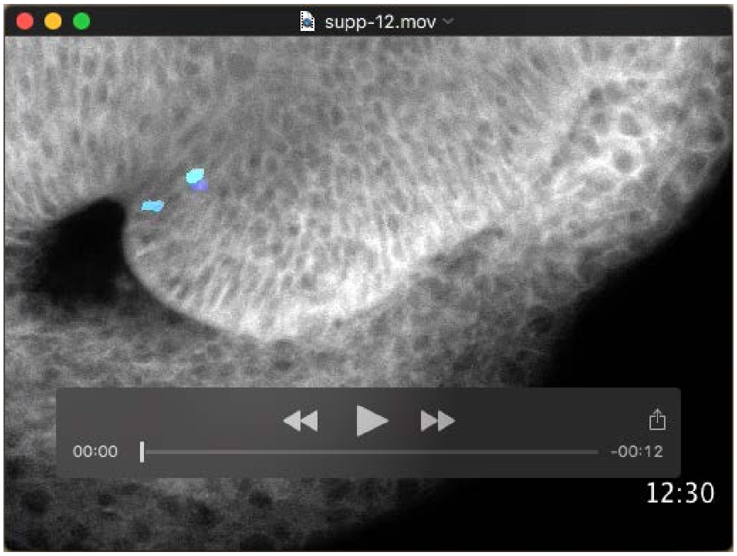

Movie 7. Related to Figure 2. *tfap2a;foxd3* double mutant nasal RPE nuclear trajectories from 12.5-24 hpf. Representative trajectories over membrane channel average (grayscale). ΔT between z-stacks, 2 minutes 30 seconds.

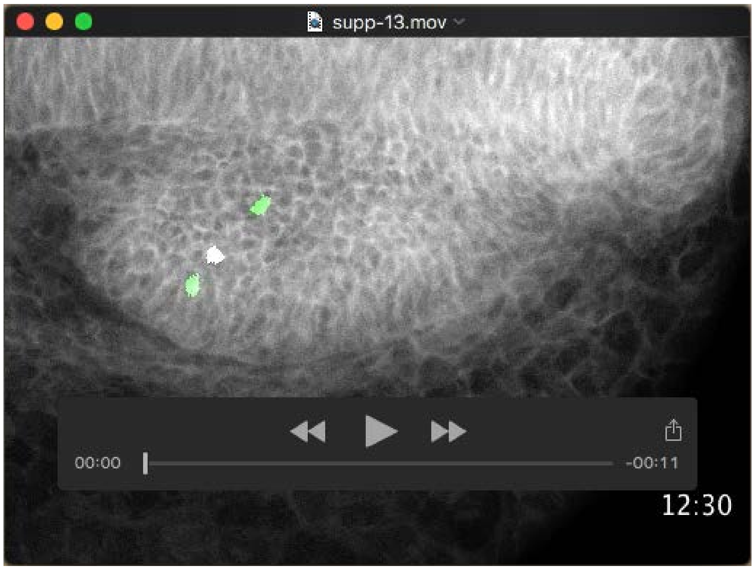

Movie 8. Related to Figure 2. Wildtype temporal retina nuclear trajectories from 12.5-24 hpf. Representative trajectories over membrane channel average (grayscale). ΔT between z-stacks, 2 minutes 45 seconds.

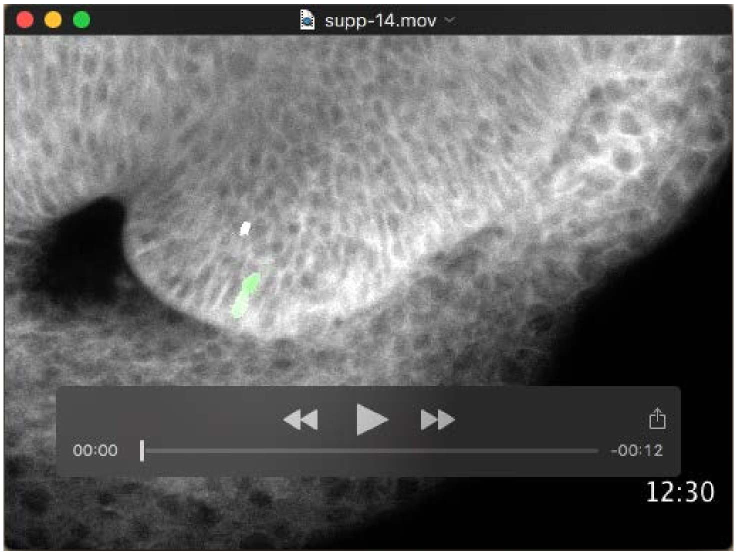

Movie 9. Related to Figure 2. *tfap2a;foxd3* double mutant temporal retina nuclear trajectories from 12.5-24 hpf. Representative trajectories over membrane channel average (grayscale). ΔT between z-stacks, 2 minutes 30 seconds.

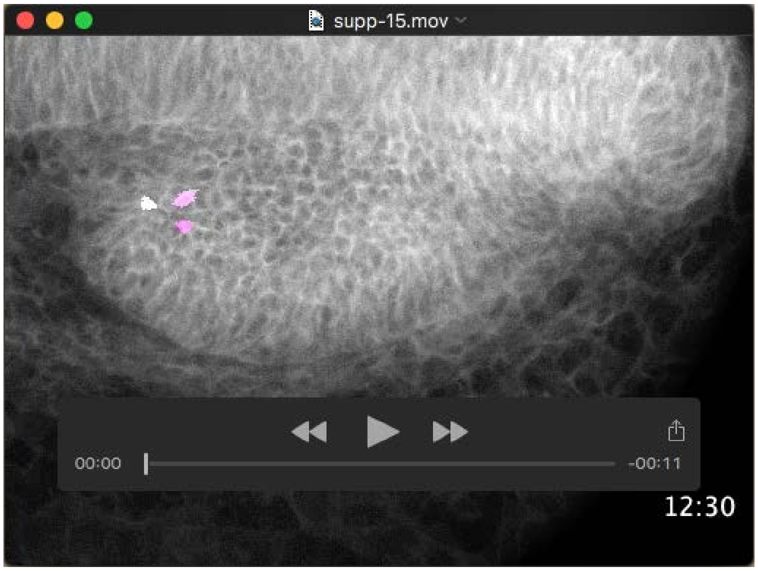

Movie 10. Related to Figure 2. Wildtype temporal RPE nuclear trajectories from 12.5-24 hpf. Representative trajectories over membrane channel average (grayscale). ΔT between z-stacks, 2 minutes 45 seconds.

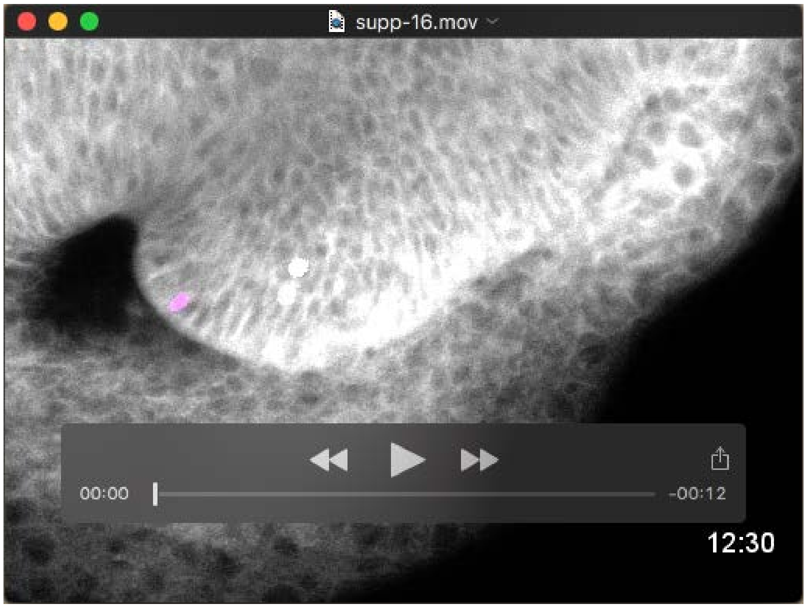

Movie 11. Related to Figure 2. *tfap2a;foxd3* double mutant temporal RPE nuclear trajectories from 12.5-24 hpf. Representative trajectories over membrane channel average (grayscale). ΔT between z-stacks, 2 minutes 30 seconds.

Table S1. RT-qPCR primers

**Gene**	**5’ (5’ – 3’)**	**3’ (5’ – 3’)**
nid1a	CATGAAAATGCTGTCTGTTTCAA	GCAACGCGTCTTTTCACGTTCTG
nid1b	CAGCCGGACTTCCACAAC	CGCTGAACTGCTGTCTGATGG
nid2a	GCCCCCATCGGAGGTCTATTTG	CCGAGTGAATTCAGCACCGGTG
nid2b	GCCTTACAGGACTCGGTTG	CGTCCAAAATCTGTCCAATACA
eef1a1l1	CCTCTTTCTGTTACCTGGCAAA	CTTTTCCTTTCCCATGATTGA

Development • Supplementary information
